# Morphogenesis of myocardial trabeculae in the mouse embryo

**DOI:** 10.1111/joa.12465

**Published:** 2016-03-29

**Authors:** Gabriella Captur, Robert Wilson, Michael F Bennett, Guillermo Luxán, Arthur Nasis, José Luis de la Pompa, James C Moon, Timothy J Mohun

**Affiliations:** ^1^ Institute of Cardiovascular ScienceUniversity College LondonLondonUK; ^2^ The Barts Heart CentreBarts Health NHS TrustLondonUK; ^3^ The Francis Crick Institute Mill Hill LaboratoryThe RidgewayLondonUK; ^4^ Intercellular Signalling in Cardiovascular Development & Disease LaboratoryCentro Nacional de Investigaciones Cardiovasculares (CNIC)Melchor Fernández AlmagroMadridSpain; ^5^ Monash Cardiovascular Research CentreMonashHEARTMonash UniversityClaytonAustralia

**Keywords:** cardiac embryology, cardiogenesis, developmental biology, non‐compaction cardiomyopathy

## Abstract

Formation of trabeculae in the embryonic heart and the remodelling that occurs prior to birth is a conspicuous, but poorly understood, feature of vertebrate cardiogenesis. Mutations disrupting trabecular development in the mouse are frequently embryonic lethal, testifying to the importance of the trabeculae, and aberrant trabecular structure is associated with several human cardiac pathologies. Here, trabecular architecture in the developing mouse embryo has been analysed using high‐resolution episcopic microscopy (HREM) and three‐dimensional (3D) modelling. This study shows that at all stages from mid‐gestation to birth, the ventricular trabeculae comprise a complex meshwork of myocardial strands. Such an arrangement defies conventional methods of measurement, and an approach based upon fractal algorithms has been used to provide an objective measure of trabecular complexity. The extent of trabeculation as it changes along the length of left and right ventricles has been quantified, and the changes that occur from formation of the four‐chambered heart until shortly before birth have been mapped. This approach not only measures qualitative features evident from visual inspection of 3D models, but also detects subtle, consistent and regionally localised differences that distinguish each ventricle and its developmental stage. Finally, the combination of HREM imaging and fractal analysis has been applied to analyse changes in embryonic heart structure in a genetic mouse model in which trabeculation is deranged. It is shown that myocardial deletion of the Notch pathway component *Mib1* (*Mib1* ^*flox/flox*^; *cTnT‐cre*) results in a complex array of abnormalities affecting trabeculae and other parts of the heart.

## Introduction

In all vertebrates the muscular walls of the heart possess a complex morphology that is most pronounced in the ventricular chambers. Whilst the outer surface of the ventricular wall is relatively smooth and covered by a thin, overlying epicardial layer, its inner surface reveals a complex structure, comprising distinct strands of myocardial muscle, termed trabeculae. These first appear as chambers delineated from the embryonic heart tube. In the mouse, this occurs approximately halfway through gestation [embryonic day 9.5 (E9.5)], whilst in the human embryo they appear at the end of the fourth gestational week (Carnegie Stage 12).

The complexity of trabecular architecture has proved challenging to capture (Sedmera et al. 2000), being variously described as ‘sheet‐like projections’ (Liu et al. 2010), ‘muscular luminar protrusions’ (Samsa et al. 2013), ‘ramifications’ (King et al. 2002), ‘ridge‐like myocardial structures’ (Staudt et al. 2014), ‘endocardial ruffles’ (Sedmera & McQuinn, 2008) and ‘finger‐like projections’ (Lavine & Ornitz, 2008) that protrude into the ventricular lumen. Such variability in description might be of little consequence if it simply reflected linguistic imprecision, or indeed indicated the range of trabecular architecture amongst different vertebrate species (zebrafish, mouse and quail in the above examples) that have been studied. In reality, it illustrates the profound difficulty of providing an accurate and meaningful description of a complex three‐dimensional (3D) structure that has been most commonly visualised through histological analysis.

There are, however, compelling reasons why an appreciation of trabecular origins and the precise nature of their architecture are important for our understanding of heart morphogenesis. In a number of vertebrate species including mammals, the trabeculated myocardium has a developmental programme of gene expression that differs from that of the adjacent myocardium of the ventricular wall (Qi et al. 2007), suggestive of unique functions. During normal development, both the arrangement and density of trabeculae undergo profound changes. In higher vertebrates it has been postulated that such remodelling may be intimately associated with formation of other cardiac structures, with suggestions including the coronary vasculature, the peripheral conduction system and the delineation of papillary muscles. Furthermore, genetic manipulation (primarily in the mouse) has demonstrated that when normal trabecular arrangement is disrupted (through either reduced or excess trabeculation), the consequences can be severe. Such mutants can show an immature ventricular conduction system (Samsa et al. 2013) or impaired myocardial contractility (Liu et al. 2010), and a number cause early‐ or mid‐embryonic lethality.

In the adult human heart, abnormal trabeculae are a component of several cardiac pathologies and congenital heart disease (Stöllberger et al. 2002). Genetically heterogeneous (Zhang et al. 2013) and identified by a variety of different diagnostic criteria (Kohli et al. 2008), trabecular abnormalities are frequently grouped together as ‘left ventricular non‐compaction’ (LVNC; Stöllberger et al. 2002). Clinical descriptions of LVNC have variously emphasised an excess of trabeculae within the ventricular chamber, changes in their overall morphology or the presence of abnormally large trabecular recesses, primarily within the apical region of the ventricle. Such abnormalities can be associated with heart failure, arrhythmia and cardiac death, and are considered a distinct class of cardiomyopathy.

For patients with LVNC suffering from impaired systolic function, prognosis is variable and treatment options limited. The challenge is to better understand the aetiology of trabecular abnormalities, in the hope that for the subset of patients with LVNC and poor prognosis, pre‐symptomatic diagnosis might become possible and risk stratification improved. In at least some cases, the ventricular walls of affected hearts show a striking resemblance to the architecture of the normal immature foetal ventricle. This has led to the suggestion that at least a proportion of LVNC could have an embryological origin; either resulting from postnatal regression to a foetal developmental programme, or perhaps the result of defects in normal ventricular development within the foetus (Milano et al. 2014).

Improvements in 3D imaging methods now make it feasible to follow development of trabecular myocardium, from its initial appearance and maturation in the embryo, to the establishment of trabecular architecture characteristic of the normal adult organ. Not only could this underpin investigations of trabecular function; if a proportion of LVNC results from pertubations in normal trabecular development, our understanding of non‐compaction disease may also benefit from such studies. Applied to experimental animals such as the mouse, better characterisation of trabecular formation and remodelling would provide the basis for identifying murine models of non‐compaction disease. These could be powerful tools for investigating its causes and may even suggest possible pre‐symptomatic diagnostic indicators.

Towards this end, here a systematic analysis of trabecular architecture in the developing mouse embryo is described using the highest 3D resolution imaging method currently available, high‐resolution episcopic microscopy (HREM; Weninger et al. 2006). The changing morphology of the trabeculae as revealed by 3D visualisation is documented, from their first appearance in mid‐gestation, through progressive remodelling during maturation of the ventricular chambers in the embryo heart. It is shown that two‐dimensional (2D) fractal analysis (Captur et al. 2014) provides a sensitive way to quantify these changes, and is capable of revealing both spatially localised and temporally restricted variations in trabecular complexity during normal development. Lastly, the combination of 3D modelling and quantitative (fractal) analysis has been applied to examine the abnormal cardiac morphology in a mutant mouse line. Genetic ablation of the Notch ligand regulator *Mib1* from the developing myocardium results in aberrant trabeculation in the embryonic heart (Luxán et al. 2013) and this effect is now quantified, and other cardiac abnormalities with which it is associated are reported.

## Materials and methods

### Preparing mouse embryo hearts for HREM

All mice (*Mus musculus*) were handled in accordance with the Guide for the Care and Use of Laboratory Animals published by the US National Institutes of Health and with the approval of the MRC National Institute of Medical Research Ethical Review Panel. Mouse embryos were obtained from NIMR:Parkes (a robust outbred strain maintained at the MRC National Institute of Medical Research) and the inbred strain, C57BL/6. Targeted inactivation of *Mib1* in C57BL/6 mouse myocardium has been previously described (Luxán et al. 2013). For approximate embryo staging, detection of a vaginal plug was taken as gestation day 0.5 (E0.5).

Embryo hearts were isolated as detailed in Supplementary methods. These were dehydrated, infiltrated with methacrylate resin and used for HREM analysis as previously described (Weninger et al. 2006). Briefly, HREM uses block‐face imaging to produce perfectly registered digital image stacks capturing the 3D architecture of the embryonic heart at high resolution. Resulting datasets comprise 1000–2000 digital, short‐axis images produced by repeated removal of 2‐μm (E10.5–E16.5) or 3‐μm (E18.5) sections (base‐to‐apex direction). These can be digitally reconstructed into detailed 3D models.

### Image processing and fractal analysis

After optimisation of grey‐scale mapping, HREM datasets were subscaled to a final size of 250 Mb for 3D volume rendering using Osirix (www.osirix-viewer.com; 64‐bit version). The subscaled data were edited to remove atria and the great vessels, resulting in image stacks comprising data from the plane of the atrioventricular valves to the apex of the heart. Approximately 100 images across each trimmed data stack were used for fractal analysis.

A fractal object is defined as a rough, fragmented or detailed geometric shape that can be subdivided into parts, each of which is a reduced copy or approximate copy of the whole, where their self‐similarity may be exact, quasi or statistical. Complex biological structures like trabeculae have quasi‐fractal properties, making them amenable to description and quantification by fractal mathematics, generating an index (the fractal dimension, FD) of their space‐filling (Captur et al. 2014; Supplementary methods).

Fractal analysis of extracted endocardial contours was performed using the standard box‐counting method where a grid of known spacing (scale) was placed over the contour image and the number of boxes that contained non‐zero pixels counted. This was repeated for multiple grids with increasing spacing. With increasing scale, the number of boxes containing non‐zero pixels decreased exponentially and the exponent was equivalent to the FD. To quantify the exponent, log–log plots of the number of boxes against scale and the gradient (−FD) was estimated using linear regression. Using this method, a total of 108 wild‐type hearts was analysed: 77 from NIMR:Parkes and 31 from C57BL/6 genetic backgrounds. Twelve *Mib1* ^flox/flox^; *cTnT‐cre* hearts were compared with nine wild‐type littermates.

### Statistical analysis

Statistical analysis and data visualisation was performed in R programming language (version 3.0.1, The R Foundation for Statistical Computing). Distribution of data was assessed on histograms and using the Shapiro Wilk test. Results are reported as mean ± standard deviation, unless otherwise stated. Categorical variables were compared using *χ* ^2^‐tests. Graphical plots are reported as group means (solid lines) and ribbons representing upper and lower 95% confidence limits. A two‐factor fully cross‐factored anova model (FD *= X|A + ε*, where epsilon signifies full replication) was used to determine statistical significance. To compare FD profiles between embryonic stages, between strains, and between mutant and wild‐type mouse populations, covariate models were constructed for the analysis of response FD to terms: *X|A*, where *A* was the fixed factor (e.g. mouse strain or embryonic stage), and covariate *X* the (numeric) relative slice position along the ventricle. Two‐sided values of *P* < 0.05 were considered significant.

## Results

### Trabecular morphology in the embryonic mouse heart

**Figure 1:**
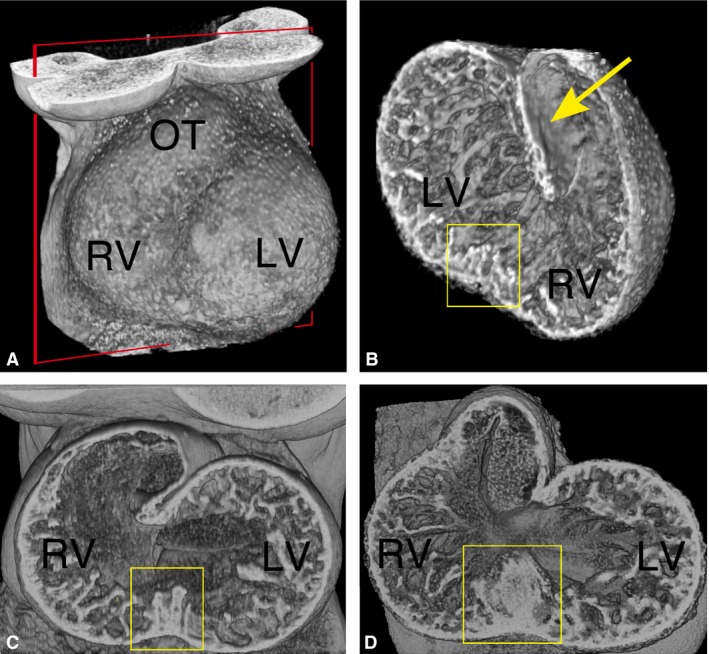
Three‐dimensional trabecular architecture in the early mouse embryo heart. (A) Thoracic region of an E9.5 embryo (ventral view), reconstructed by volume rendering from HREM data. The ballooning left and right ventricular chambers (LV and RV) are clearly visible through the thin pericardium, as is the outflow tract (OT). The plane of erosion for (B) is shown (red box). Scale bar: 200 μm. (B) Erosion from the dorsal side reveals a mesh‐like network of trabeculae throughout the common ventricular chamber, which contrasts with the smooth luminal surface of the outflow tract (yellow arrow). The trabeculae appear raised and more closely interconnected at the site of the future IVS (yellow box). Scale bar: 200 μm. (C and D) Three‐dimensional models of the heart at E10.5 and E11.5, respectively. Ventro‐inferior erosion through the ventricular chambers towards the atrioventricular valve reveals progressive coalescence of trabeculae to form the base of the IVS (boxed). Scale bar: 200 μm.

Three‐dimensional models obtained from HREM data reveal the architecture of ventricular trabeculae as the normal mouse heart develops. The beginnings of trabeculation can first be seen at E9.5 as the prospective ventricular chambers balloon out from the common ventricular portion of the looped heart tube (Fig. 1A,B). At this early stage, the trabeculae form a meshwork, with no discernable pattern in the orientation or branching of the trabecular strands either with respect to each other, or to the surface of the ballooning ventricular wall. Between the prospective apical portions of the developing left and right ventricular (LV and RV) chambers, the trabeculae become noticeably raised and prominent, marking the beginnings of the interventricular septum (IVS). Over the next 2 days, the base of the IVS becomes clearly established, its morphology suggesting progressive coalescence by the trabeculae (Fig. 1C,D).

**Figure 2:**
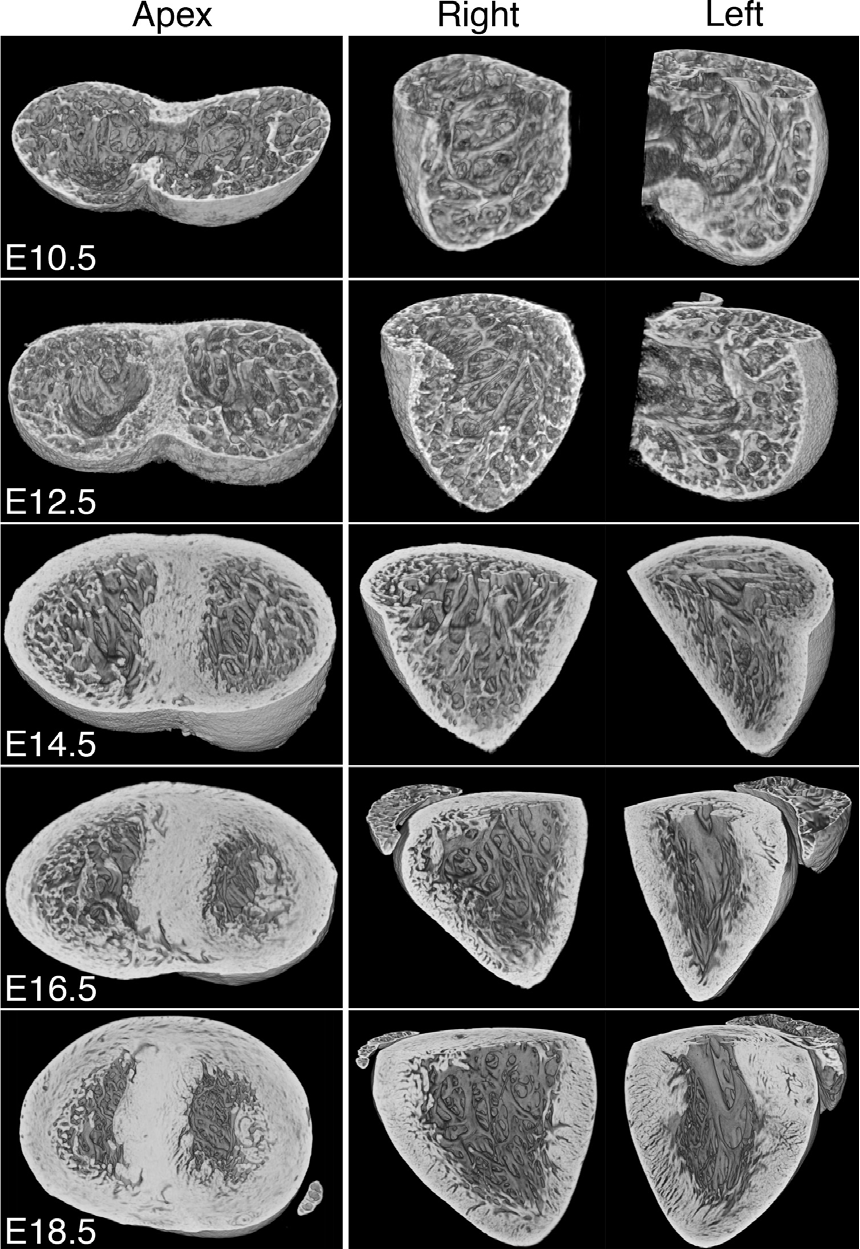
Changes in trabecular morphology during embryo development. Three‐dimensional models from HREM data for isolated hearts at successive stages of embryonic development. Models have been eroded in the short axis to provide views of the trabecular meshwork in the apex of the ventricles. Erosion in the plane of the IVS provides views of the left and right ventricular (LV and RV) chambers. Scale bars: 200 μm (E10.5); 500 μm (E18.5).

As ventricular septation proceeds (E10.5–E14.5), the trabecular mesh becomes more densely packed and complex. Little difference can be seen between LV and RV in either mesh density or the thickness of the constituent trabecular strands even in their apical regions (Fig. 2). From E12.5, in both chambers the outer, compact portion of the ventricular wall thickens several‐fold, as does the IVS. The arrangement of the trabeculae shows an increasing alignment, broadly in an apex‐to‐base orientation, a transformation that is striking by E14.5 (Fig. 2).

**Figure 3:**
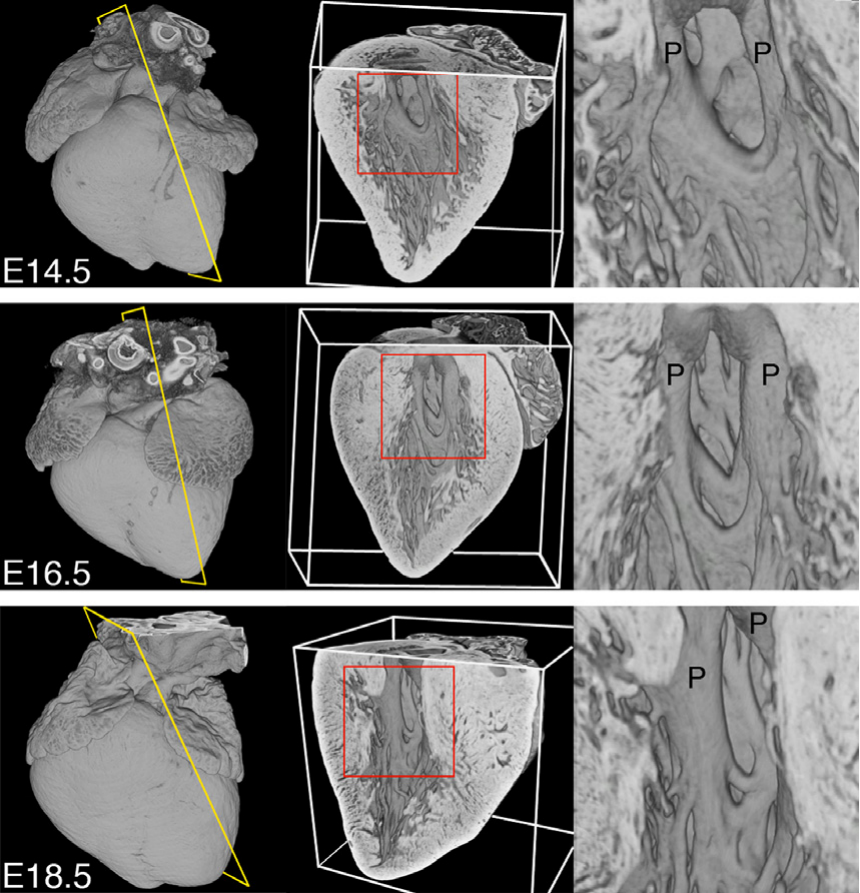
Trabeculae support the developing papillary muscles. Three‐dimensional models digitally eroded along the indicated planes (yellow) to view papillary muscles (P) of the LV at E14.5, E16.5 and E18.5 (views from the right; regions enlarged are shown in red). The trabecular mesh merges to form the roots of the developing papillary muscles, and this intimate arrangement is maintained throughout embryonic development. Scale bar: 500 μm (E18.5).

From E14.5, thickening of both septal and free walls of the ventricular chambers continues, but complexity of the trabecular meshwork appears to become somewhat reduced. In each chamber, trabecular strands have merged together to form the base of the growing papillary muscles (Figs 2 and 3), the arrangement of which now distinguishes LV from RV chambers (compare Fig. 3 with Fig. S1). A second distinguishing difference between chambers is evident in the surface of the left and right faces of the IVS. Digital ‘casts’ of lumen volumes clearly illustrate that much of the RV lumen septal face is relatively smooth, in contrast to the much more trabeculated septal surface of the LV lumen (Fig. S2; Movie S1).

Importantly, despite progressive alignment of the major trabecular strands during development, they remain extensively cross‐linked in a mesh, an observation first noted two decades ago from scanning electron microscopy (Sedmera & Thomas, 1996), but subsequently often overlooked. At no stage do the trabeculae show a 3D morphology that can be described as ‘finger‐like’ (with concomitant bases and tips; cf. Runge & Patterson, 2007; Park et al. 2013), although individual 2D sections through the trabecular mesh can mistakenly be interpreted to suggest such an arrangement.

### Developmental changes in trabecular morphology

Preliminary studies established that variations of up to 20 ° in the precise 2D imaging plane had little effect on 2D FD measurements and would not therefore compromise inter‐sample comparisons (Supplementary methods; Figs S3 and S4). Taking advantage of the very large number of serial section images captured by HREM, hearts were analysed at successive stages of development, measuring the 2D FD value along the sectioning axis and tracking how this changes from completion of ventricular septation (E14.5) to birth (E18.5).

**Figure 4:**
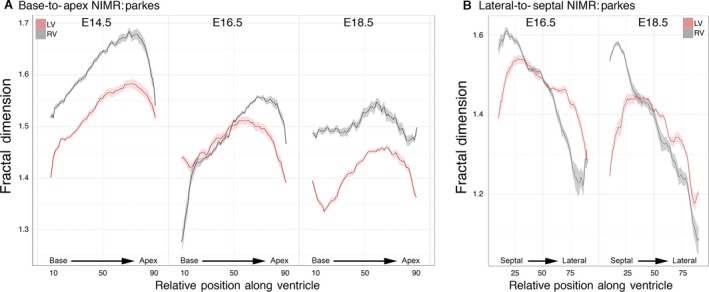
Fractal‐based quantification of trabecular complexity during normal development. The FD was calculated from HREM image data obtained with the outbred mouse strain, NIMR:Parkes. Fractal analysis is a method of quantifying complex geometric patterns in biological structures. The resulting FD is a unitless measure index of how completely the object fills space, increasing with increased structural complexity. FD was measured using a box‐counting method on consecutive HREM slices of the embryo hearts in two orientations: base‐to‐apex and lateral‐to‐septal. (A) Base‐to‐apex profile of LV and RV trabecular development (pink and grey, respectively) at stages E14.5 (LV:* n* = 15; RV:* n* = 11); E16.5 (LV:* n* = 13; RV:* n* = 11) and E18.5 (LV:* n* = 14; RV:* n* = 12). The fractal signatures of the morphological RV and LV are relatively similar at E14.5 (*P* = 0.951), but their patterns diverge as development proceeds, and by E18.5 are markedly different (*P* < 0.0001). (B) The LV and RV chambers from E16.5 (*n* = 23) and E18.5 hearts (*n* = 20) were digitally resliced in an orthogonal plane, from lateral‐to‐septal surface. At E16.5 and E18.5 note the differing lateral‐to‐septal patterns of FD between LV and RV (pink and grey, respectively) and the consistently higher FD value in the lateral wall of the RV compared with the equivalent position in the LV (*P* < 0.0001 both stages). (Solid lines: mean FD value; shaded ribbons: 95% confidence interval. Only FD values for slices from 10% to 90% of Base‐to‐apex or lateral‐to‐septal axis are shown.)

Visual inspection of 3D models from embryonic hearts suggests a decrease in trabecular complexity concomitant with the thickening of the compact myocardial wall (Figs 2 and 3). Slice‐by‐slice fractal analysis of hearts from the outbred NIMR:Parkes strain confirms this change, with absolute FD values across all regions of the ventricle decreasing progressively with development (Fig. 4A). The greatest transition was observed between E14.5 and E16.5, with less occurring closer to term. At each stage, FD values show a characteristic and reproducible profile, complexity being highest in the apical half of both LV and RV chambers compared with the basal half (e.g. mean basal FD vs. mean apical FD at E14.5: for LV, 1.495 vs. 1.568, *P* < 0.001; for RV, 1.581 vs. 1.657, *P* < 0.001).

For 24 of the hearts, the image series were digitally re‐sectioned orthogonally, generating a virtual section plane parallel to the IVS. With these, the distribution of trabecular complexity was evaluated along an axis from lateral to septal walls. From this it was clear that at E16.5 and E18.5 (Fig. 4B), the lateral luminal wall of both ventricles exhibits significantly greater trabecular complexity when compared with the septal surface, an observation strikingly illustrated by 3D models of ventricular lumen volumes (Fig. S2; Movie S1).

### Comparison of LV and RV

Fractal analysis revealed a clear and consistent difference in the degree of trabecular complexity between the LV and RV, a difference that is not obvious from visual inspection. At each stage, the RV profiles are consistently higher in FD value in the apical half of the chambers, and a similar difference is evident in the basal half at E14.5 and E18.5. In addition to these quantitative differences, the shape of the LV and RV fractal profiles increasingly diverge as development proceeds. The notable difference in surface of the luminal septal wall between LV and RV chambers is clearly seen in the consistently lower RV FD profile in the septal half of the lateral‐to‐septal plots (Fig. 4B). This contrasts with the higher RV values for the luminal surface of the free (lateral) wall compared with the LV. Together, the orthogonal fractal plots localise the greater complexity of the trabecular network in the RV to the apical and free wall regions of the chamber.

### Strain background affects trabecular profile

**Figure 5:**
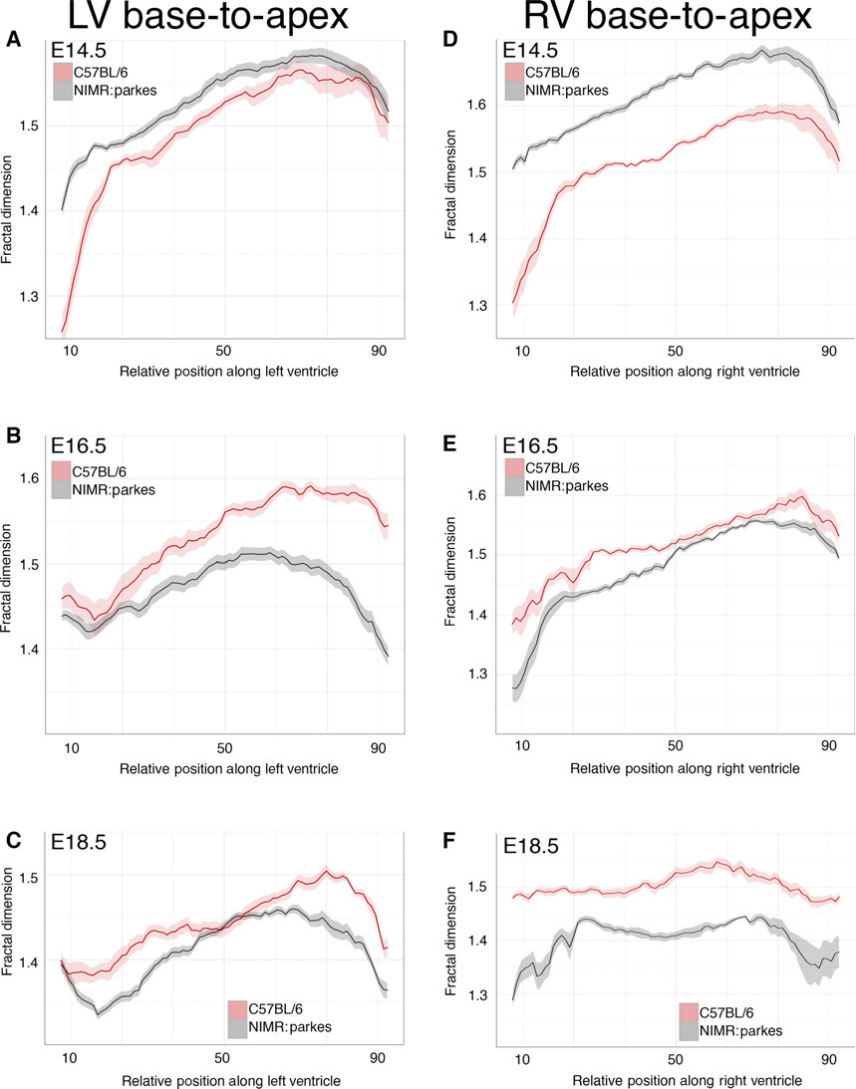
Strain differences in trabeculation detected by fractal analysis. Comparison of base‐to‐apex fractal profile of LV and RV trabeculae between the outbred strain, NIMR:Parkes (grey) and the inbred strain, C57BL/6 (pink). (A–C) LV data: E14.5 (*n* = 28); E16.5 (*n* = 21); E18.5 (*n* = 24). (D–F) RV data: E14.5 (*n* = 19); E16.5 (*n* = 18); E18.5 (*n* = 18). Also see Fig. S3. (Solid lines: mean FD value; shaded ribbons: 95% confidence interval. Only FD values for slices from 10% to 90% of base‐to‐apex are shown.)

Next it was tested whether mouse strain background had an impact on fractal profile, comparing the outbred NIMR:Parkes strain with the commonly used inbred line, C57BL/6. Base‐to‐apex profiles of FD for both LV and RV (Fig. 5) are largely similar between strains as development proceeds, but some significant differences are also evident. Notably, at E16.5 (Figs 5B and S5), fractal complexity in the apical half of the LV is greater in C57BL/6 than in NIMR:Parkes (*P* < 0.0001). By E18.5, LV trabecular profiles between the two strains are less dissimilar (*P* = 0.093), although the apical region continues to show relatively more complexity in C57BL/6. For the RV, at E14.5, fractal complexity is greater in NIMR:Parkes compared with C57BL/6 (*P* < 0.0001) but beyond this, at E16.5 and more so at E18.5, C57BL/6 begins to exhibit significantly greater RV fractal complexity compared with NIMR:Parkes (*P* = 0.001 and *P* < 0.0001, respectively). These data suggest a subtle difference in temporal pattern of trabecular morphogenesis between the two strains, imperceptible by visual inspection but readily detected by fractal quantitation.

### Quantification of abnormal trabeculation during embryonic development

Finally, HREM was used to examine a recently described mouse model that exhibits an abnormal increase in ventricular trabeculation in the embryonic heart. *Mib1* is an E3 ubiquitin ligase responsible for Notch1 activation in the developing trabeculae where it carries out Notch ligand ubiquitination. In *Mib1* ^flox/flox^; *cTnT‐cre* mouse mutants, targeted ablation of *Mib1* in the developing myocardium leads to impaired Notch1 activity, resulting in a ‘non‐compaction’ phenotype (Luxán et al. 2013).

**Figure 6:**
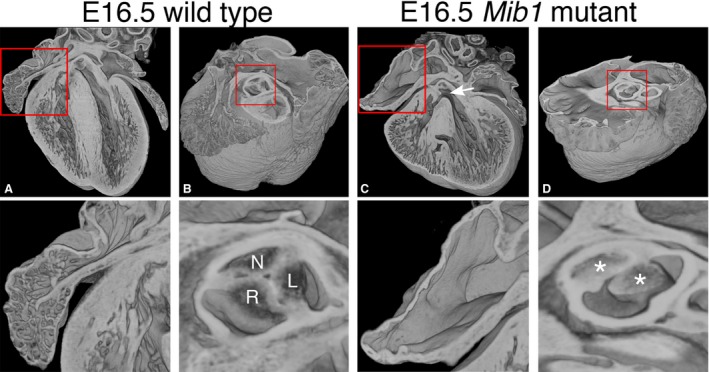
Morphological abnormalities in the *Mib1* mutant heart. Three‐dimensional volume renderings of E16.5 *Mib1* mutant and wild‐type, sibling hearts. Models are eroded from the anterior (A and C) to give a four‐chamber view, and from the dorsal side (B and D) at the level of the developing aortic valve. Regions highlighted are shown (red boxes). Note the grossly aberrant trabecular arrangement, thin ventricular wall and ventricular septal defect (arrow) in the *Mib1* mutant (C). Wild‐type left and right atria have normal pectinate muscle morphology, while there is near‐complete effacement in *Mib1* mutants (compare A with C, highlighted). The developing aortic valve in the *Mib1* mutant is abnormal, in this case the left and non‐coronary leaflets (highlight D, asterisks) lying well above a reduced sized right leaflet. Compare with the normal trifoliate (left, right and non‐coronary) leaflet arrangement (highlight B, labels L, R and N, respectively). Scale bar: 500 μm (E16.5).

**Figure 7:**
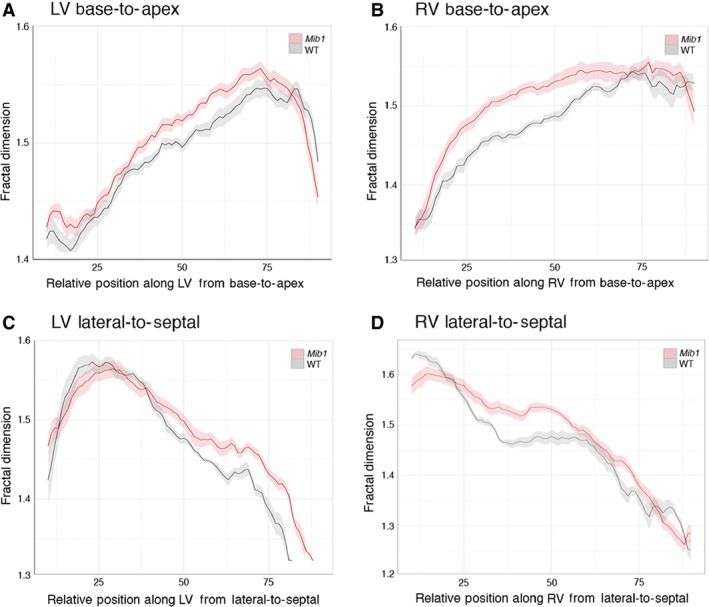
Fractal analysis of *Mib1* mutant hearts. Base‐to‐apex and lateral‐to‐septal fractal dimension (FD) plots comparing E16.5 *Mib1* mutant (*Mib1* ^flox/flox^; *cTnT‐cre*) hearts (pink) with wild‐type littermates (grey). Note that for both LV and RV, fractal analysis detects significant and consistent patterns of variation between mutant and control hearts. Also see Fig. S5. (Solid lines, mean; shaded ribbons, 95% confidence intervals).

At E16.5, visual inspection of HREM datasets confirmed that all *Mib1* ^flox/flox^; *cTnT‐cre* embryos had a dilated heart with abnormally thin compact myocardium and an unusually dense and extensive trabecular network (Fig. 6, compare A and C). This was true for both LV and RV, with the RV lumen being almost completely occluded by the trabecular mesh. Fractal analysis provided a more detailed and quantitative assessment of these changes, with the profile of the RV (base‐to‐apex) differing more profoundly than the LV from the equivalent profiles of wild‐type littermates (*P* = 0.036 compared with 0.166; Fig. 7A,B). The *Mib1* mutants also showed a significant divergence in lateral‐to‐septal profile of trabecular complexity (Fig. 7C,D), particularly in the LV (LV, *P* = 0.0001; RV, *P* = 0.661).

In addition to aberrant trabeculation, HREM imaging revealed additional abnormalities in the morphology of hearts from embryos of the myocardial *Mib1* deletion mutant (Fig. 6): nine out of the 12 mutants showed perimembraneous or muscular ventricular septal defects (e.g. Fig. 6C); eight of the 12 mutants exhibited right and left atrial myocardial hypoplasia, characterised by a marked reduction or complete absence of pectinate muscles (Figs 6C and S7). In addition, many of the mutants showed abnormalities in the arrangement of the developing aortic valve leaflets. These varied in severity and, whilst the overall trifoliate arrangement remained recognisable, individual leaflets were abnormally proportioned, with one often positioned below the other two along the axis of the vessel (Fig. 6, compare B and D; Movie S2).

## Discussion

In recent years, considerable progress has been made in identifying signalling pathways and cellular interactions that underlie formation of trabeculae in the mammalian heart (for review, see Samsa et al. 2013), but there has been little comparable advance in understanding of the tissue morphology that such developmental pathways produce. In part this may be attributed to the reliance upon 2D analysis for extrapolating 3D structure, an approach prone to yielding misleading results; in part it also reflects the very complexity of trabecular architecture.

The absence of accurate models of changing trabecular morphology not only impacts on our understanding of heart development, it may also compromise progress in understanding the aetiology of non‐compaction disease. Without a clear picture of normal trabecular structure and the changes it undergoes during embryonic stages, it is difficult to identify any but the most extreme examples of trabecular disruption during development (Supplementary Table 1). Amongst laboratory animals such as the mouse, these are unlikely to be very fruitful as models for investigating human non‐compaction disease because their phenotype is so extreme. The challenge is therefore twofold; to establish an accurate description of trabecular morphogenesis in the normal mouse embryo heart, and to use this as a baseline to identify mutants showing more subtle alterations in trabecular arrangement that may better model human non‐compaction disease.

Here it was shown that by using HREM, and the detailed 3D modelling it facilitates (Mohun & Weninger, 2011), it is possible to visualise trabecular morphology and follow the changes in trabecular architecture that occur during embryonic heart development. Crucial to this is the ability to isolate embryonic hearts in diastole, when the full extent of trabecular arrangement within the ventricular lumens can be visualised. Equally important is the removal of any remaining blood, as this confounds accurate tissue imaging. Both can be achieved relatively easily, using procedures that do not apparently affect overall cardiac size or architecture. With 2‐μm isotropic volume datasets, from their formation at mid‐gestation (E9.5), it is found that trabeculae in the mouse heart form a complex mesh, an arrangement that is maintained despite all subsequent remodelling during development. This is quite different from the trabecular organisation reported for chick embryos, in which an initial, more ordered radial array appears to be replaced by a more spiral architecture (Sedmera et al. 1997). The murine trabecular mesh acquires a more aligned character as development proceeds, but the extent to which the resulting structure is stereotypical or stochastic remains to be investigated. Quantitation of parallelism may offer additional insights into the process of trabeculation and compaction. Certainly the orientation of this alignment matches that of the developing papillary muscles, and it is striking how the trabeculae form a root‐like network at the base of these muscles. In the adult human heart, trabeculae are believed to transmit the pressures exerted by the mitral ring and tendinous chordae from the papillary muscles, towards the heart's apex and ventricular wall (Axel, 2004; Filipoiu, 2014), and the manner in which the bases of papillary muscles associate with trabeculae in the mouse embryo heart suggests a likely similar role.

In human hearts, LV and RV show differences in the extent and ‘coarseness’ of trabeculae, a difference that can be helpful in assessing extreme pathological abnormalities in chamber arrangement. From 3D modelling it is also striking that the mouse embryo heart does not share such obvious differences. Three‐dimensional models also reveal that in the early embryo (E9.5), trabeculae along the sulcus formed between the LV and RV become raised and more densely packed, marking the base of the future IVS. Localised coalescence of trabeculae appears to underlie initial formation of the IVS, but the extent to which this mechanism accounts for subsequent septal growth is currently unclear.

By its nature, the extent and morphology of a 3D mesh is difficult to quantify. None of the established clinical methods for assessing trabecular non‐compaction satisfactorily resolve this problem, nor are they readily applicable to murine embryo hearts. Of particular note is the extraordinary variability that would be obtained by the frequently adopted clinical approach of measuring the ratio of compact to trabecular layers. Certainly with embryonic stage mouse hearts, such ratios are so dependent upon image plane and measurement point (Fig. S7) that they are valueless. The current data have not been histologically correlated, but they demonstrate that fractal analysis provides a viable alternative solution, offering an objective measure of trabecular mesh complexity by assessing the 2D FD of individual HREM images. For trabecular quantification, it has been argued elsewhere that lacunarity is not as sensitive or helpful an approach as FD (Captur et al. 2015). In addition to providing a quantitative index of trabecular complexity, the current results show that this approach also detects subtle variations in trabecular architecture, expressed in variations within the profiles of FD. Perhaps most importantly, the fractal approach permits developmental changes in trabeculation to be measured, thereby providing an objective index for assessing both gross and subtle trabecular abnormalities. Without a convincing mouse model of clinical non‐compaction disease, rather than the existing array of extreme disruptions to cardiac morphology, it is not possible yet to assess the ability of FD in detecting non‐compaction imperceptible by qualitative analysis. However, its ability to detect regional, left : right and strain variations in trabecular complexity within normal hearts suggests that it may indeed have sufficient sensitivity.

A number of different methods have been used clinically to assess trabecular structure and diagnose non‐compaction in the human heart. These vary widely in approach, and their reliability has been questioned by the finding that they can yield inconsistent results (Kohli et al. 2008). Of these, measurement of the ratio between trabecular and compact myocardial wall thickness (Petersen et al. 2005) is particularly attractive as it offers the possibility of a quantitative measure for following trabecular morphology in the developing mouse embryo. However, it is readily apparent that variation in trabecular mesh morphology around the ventricular chamber and the critical dependence of measured values on 2D section plane together render this method valueless for embryo data (Fig. S7). As an alternative, fractal analysis was used (Captur et al. 2014) to provide a quantitative assessment of trabecular complexity in a manner that was not compromised by such variations.

The observations of a dynamic LV base‐to‐apex and lateral‐to‐septal pattern of fractal complexity could suggest an influence of LV inflow and outflow blood circuits on the development of the trabeculated myocardium. Recent human data (Carlhäll & Bolger, 2010) using time‐resolved 3D phase‐contrast cardiovascular magnetic resonance (CMR) have provided insights into the normal flow of blood in the functioning LV cavity, showing how this is comprised of direct and retained components. Theoretical poroelastic cardiac models of the embryonic chick heart (Yang et al. 1994) have suggested that similar circuits are in operation in the developing ventricular cavities. In the adult human heart, the slow‐moving, low kinetic energy residual blood volume has been shown to settle along the mid‐to‐apical inferolateral aspect of the LV and it fills the apical cap for the whole duration of the cardiac cycle. The current fractal data suggest that these are also the areas of greatest trabecular persistence in the developing mouse heart. A plausible hypothesis is that blood flow‐related transmural stresses during embryogenesis may be influencing LV trabecular patterning. The observation of a consistent base‐to‐apex profile of trabecular complexity in both ventricles of the mouse embryo heart is similar to observations of the adult human LV structure, studied by CMR (Captur et al. 2014).

Fractal dimension profiles indicate a subtle but consistent difference in the trabecular mesh of the LV and RV of the mouse embryo, whether assessed along base‐to‐apex or lateral‐to‐septal axes. Differences have previously been reported between the two ventricles in the chick embryo, with shorter, stick‐like trabeculae in the RV compared with coarser, thicker equivalents in the LV (Sedmera et al. 1997). In humans, differences in the trabecular thickness have been used as a diagnostic feature to distinguish morphologically LV from RV in the setting of abnormal cardiac morphology, but it is debatable whether a comparable difference can be observed from qualitative inspection of the adult mouse heart. Certainly, prior to birth, no such difference is visually evident from 3D models of heart structure, but fractal analysis now confirms that a measurable difference does indeed exist, 2 days before term.

Mouse genetic background is known to affect normal physiology as well as being an important determinant of pathological phenotype. For example, it impacts on cardiac response to fibrosis, ischaemia‐reperfusion injury, and to pathological states like insulin resistance, atherosclerosis and myocardial hypertrophy (Walkin et al. 2013; Vaillant et al. 2014). Similarly, it can significantly alter embryonic and adult phenotypes obtained as a result of gene mutation. The current finding of subtle differences in the fractal profiles obtained from outbred and inbred mouse strains demonstrates both the sensitivity of this approach and the experimental importance of ensuring appropriate strain controls in any quantitative assessment of trabecular morphology.

The current models of the developing mouse heart clearly show that between E12.5 and E14.5 the compact layer of the ventricular wall expands dramatically and, over the same period, the complexity of the trabecular mesh also increases. It is difficult to reconcile these observations with the suggestion that growth in thickness of the compact layer results from ‘compaction’ of trabeculae. Even during later development (E14.5–E18.5), when trabecular complexity indeed reduces, the increase in compact wall volume appears disproportionate compared with the decline in trabeculae. Only a combination of 3D trabecular cell lineage mapping and proliferation studies will resolve whether ‘compaction’ (and by implication ‘non‐compaction’) is an unfortunate misnomer or an accurate description of developmental events.

In the human heart, pathological abnormalities in trabeculation have been identified using a variety of diagnostic criteria. However, it is unclear to what degree these monitor different facets of a common phenotype or the extent to which non‐compaction encompasses a spectrum of abnormalities with distinct aetiologies. In at least a proportion of cases, the affected hearts have an unusually extensive trabecular mesh, an arrangement abnormal for the adult heart but reminiscent of the morphology found prior to birth in both humans and mice. It is notable that in such cases, there is little compelling evidence of any significant overall diminution in compact layer thickness. This is quite unlike the profound thinning of the compact layer in many mouse genetic mutants reported to exhibit ‘non‐compaction’ or ‘hypertrabeculation’, including the *Mib1* mutant studied here.

Three‐dimensional models of *Mib1* mutant hearts and the accompanying fractal analysis graphically illustrate the abnormal trabecular density in the mutant embryos. They also demonstrate the divergence between the abnormalities in this mutant and those identified in clinical cases of LVNC. Most obviously, the increase in trabecular mesh complexity coincides with a dramatic reduction in compact wall thickness. Furthermore, the increased trabecular complexity appears qualitatively more extreme in the RV rather than the LV, an observation from 3D models that is confirmed by fractal‐based quantitation. The current models also revealed a profound abnormality in atrial morphology, with a large reduction or complete absence of pectinate muscles that normally give a trabecular‐like morphology to the atrial appendages (Fig. S6). Most *Mib1* mutant hearts also showed a large perimembranous ventricular septal defect, with additional septal defects in the muscular portion of the IVS. Indeed, in several, the base of the IVS bore numerous deep clefts, some of which connected to provide communication between LV and RV lumens.

Genetic analyses by other groups (Nakashima et al. 2014) have already implicated Notch signalling in diverse aspects of murine heart morphogenesis, its disruption for example impacting upon outflow tract development, proliferation of the atrial chamber myocardium, the atrioventricular node (Rentschler et al. 2011) and resulting in ventricular septal defects (Niessen & Karsan, 2008; Luxán et al. 2013; Saravanakumar & Devaraj, 2013). The current data provide striking confirmation of this, the *Mib1* mutants exhibiting multiple malformations in cardiac morphology. Because human *Mib1* mutations have been associated with some cases of autosomal‐dominant familial LVNC (Luxán et al. 2013), the current studies suggest that Notch signalling alterations may cause a specific type of ‘non‐compaction’, which may also be associated with other cardiac abnormalities.

The Notch pathway is also important for aortic valve formation, and indeed mutations in the human Notch1 locus have been linked to aortic valve disease, most notably bicuspid aortic valve. A proportion of the *Mib1* mutants show both abnormal trabecular architecture and severely malformed aortic valves, and it is for future studies to establish if this combination has an as‐yet unrecognised human disease corollary.

## Funding

This work was supported by funding to TJM from the Medical Research Council (U117562103); to JCM by the Higher Education Funding Council for England; to JLdlP and GL by grants SAF2010‐17555 and SAF2013‐45543‐R from the Spanish Ministry of Economy and Competitiveness (MINECO); and to GC by a research fellowship at the University College London Biomedical Research Centre from the UK National Institutes of Health Research Cardiometabolic Programme.

## Author contributions

JCM, TJM and GC developed the concept and approach; GC performed the fractal experiments and data analysis; TJM led the HREM dissections; TJM, GC and JCM prepared the paper prior to its submission; RW provided statistical and expert advice; MB assisted with the HREM of mouse embryos; GL and JLdlP provided the *Mib1* mutant and wild‐type littermates, and expert guidance; AN assisted with image processing after HREM.

## Conflicts of interest

The authors declare no competing financial interests.

## Supporting information


**Data S1.** Methods.

**Fig. S1.** Papillary muscles of the right ventricle.

**Fig. S2.** Volumetric models of right and left ventricular lumens.

**Fig. S3.** Comparing E16.5 wildtype hearts across two strains.

**Fig. S4.** Effect of relative section plane on fractal dimension profile.

**Fig. S5.** 3D models of an E16.5 *Mib1* mutant (*Mib1^flox/flox^; cTnT‐cre*) embryo heart and a wildtype sibling (antero‐lateral views).

**Fig. S6.** Comparison of HREM section images through the atria of an E16.5 *Mib1* mutant (*Mib1^flox/flox^; cTnT‐cre*) embryo heart and a wildtype sibling.

**Fig. S7.** Virtual images generated from E14.5 and E18.5 HREM datasets (Panels A and B, respectively; NIMR:Parkes strain).

**Movie S1.** Ventricular luminal models of embryonic wildtype mouse heart at E14.5.

**Movie S2.** HREM image stack of an E16.5 *Mib1* mutant embryo heart.
