# Supplementary material for: Morphogenesis of myocardial trabeculae in the mouse embryo
Source: J Anat. 2016 Mar 29;229(2):314–25. doi: 10.1111/joa.12465 (PMC4948049; doi:10.1111/joa.12465)
Supplement: Supplementary file 1 — Data S1. Methods. [file JOA-229-314-s001.docx]

**METHODS**

Removal of blood from embryo hearts

In order to minimise retention of blood in embryo hearts, harvested embryos were first agitated in phosphate buffered saline (PBS) solution at 37°C containing heparin for approximately 15 minutes (min), umbilical vessels being repeatedly clipped to allow blood to be pumped out. Potassium chloride was then added (final 50mM) to ensure that hearts arrested in diastole. Hearts (including attached lungs and thymus) were then isolated, washed briefly in fresh PBS and after removal of at least one lung lobe, samples were fixed for 30 min in fresh 4% paraformaldehyde at 4°C. To remove remaining blood within the heart chambers, samples were then washed in repeated changes of distilled water over 30-60min at room temperature with constant agitation (roller). The resulting osmotic shock lysed any remaining blood within the heart chambers without any alteration to heart structure as assessed by histology. After overnight fixation in 4% paraformaldehyde (4°C), hearts were dissected away from associated lung, thymus and pericardial tissue prior to dehydration and embedding in methacrylate resin (Mohun et al, 2012). Samples were positioned during embedding to ensure relatively reproducible base-to-apex sectioning during the HREM imaging process.

Fractal analysis of HREM images

HREM images were converted from 8 bit greyscale to binary and divided to separate left and right ventricular chambers using Osirix (Rosset et al, 2004) software 3D segmentation. Datasets were subsampled to yield approximately 100 images for each ventricular chamber. Luminal contours were then extracted from binary images using MATLAB® (R2012b (The MathWorks Inc., Natick, MA, USA), with in-house code for Otsu binarization (Otsu et al, 1979), a region-filling batch action that converted the image background to white, data inversion and a final edge-detection.

For fractal analysis, background pixels were set to black to permit box-counting of white foreground pixels only with smoothing filters disabled. A bounding box oriented with the *x, y* axes of the image plane was applied to each digital image to determine the relative size (pixel diameter) of the largest grid box in calculating the series of sampling sizes and scale for box counting. We determined a scaling rule for the relationship between box count and box size, following on from the assumption that these corresponded to detail and scale (ɛ) according to the equation: $\boldsymbol{FD=}\boldsymbol{lim}_{\boldsymbol{\varepsilon\to0}} \left[ {\log\boldsymbol{N\varepsilon}}/{\log\boldsymbol{\varepsilon}} \right]$, where the limit is found as the slope of the regression line for the plot of the relationship between change in count, with change in grid calibre. We replicated the analysis on the same edge image 4 times, using the same series of grid calibres but 4 different and randomly determined coordinates for starting grid positions. The final FD for any single ventricular slice was calculated as the mean of these 4 values. Results to the nearest third decimal place were automatically labelled and parsed into a comma separated variable summary data file that underwent statistical analysis. Plots of FD profile were obtained for images covering 10-90% of the image stack to minimise artefacts arising from the presence of valve leaflets or the compact ventricular wall.

Testing the effect of image plane on FD measurements

To test whether small variations in precise image plane might compromise inter-sample comparisons, we compared 2D FD plots for samples before and after digital reslicing with a 10° and 20° tilt in section plane. We used a two-factor fully cross-factored ANOVA model to test for differences. Results for the LV at both E14.5 and E18.5 (Fig. S4) show that the fractal method is robust to such variations in section plane. Comparing tilted planes with the original, at E14.5 *P* values are 0.230 and 0.066 for 10° and 20°, respectively; at E18.5, the equivalent values are 0.658 and 0.220. Similar reliability was found with the RV (data not shown).

REFERENCES FOR SUPPLEMENTARY METHODS

Mohun, T.J., Weninger, W.J., 2012. Embedding embryos for high-resolution episcopic

microscopy (HREM). Cold Spring Harb. Protoc. 6, 678–680.

Otsu, N., 1979. A threshold selection method from gray-level histograms. IEEE Trans. Syst.

Man. Cybern. 9, 62–66.

Rosset, A., Spadola, L., Ratib, O., 2004. OsiriX: an open-source software for navigating in

multidimensional DICOM images. J. Digit. Imaging. 17, 205–216.

**SUPPLEMENTARY FIGURE LEGENDS**

**Figure S1.** **3D trabecular architecture in the early mouse embryo heart.**

Panel A: Thoracic region of an E9.5 embryo (ventral view), reconstructed by volume rendering from HREM data. The ballooning left and right ventricular chambers (LV and RV) are clearly visible through the thin pericardium, as is the outflow tract (OT). The plane of erosion for panel B is shown (red box).

Panel B: Erosion from the dorsal side reveals a mesh-like network of trabeculae throughout the common ventricular chamber, which contrasts with the smooth luminal surface of the outflow tract (yellow arrow). The trabeculae appear raised and more closely interconnected at the site of the future interventricular septum (yellow box).

Panels C and D: 3D models of the heart at E10.5 and E11.5, respectively. Ventro-inferior erosion through the ventricular chambers towards the atrioventricular valve reveals progressive coalescence of trabeculae to form the base of the interventricular septum (boxed).

**Figure S2.** **Changes in trabecular morphology during embryo development.**

3D models from HREM data for isolated hearts at successive stages of embryonic development. Models have been eroded in the short axis to provide views of the trabecular meshwork in the apex of the ventricles. Erosion in the plane of the interventricular septum provides views of the left and right ventricular chambers.

**Figure S3.** **Trabeculae support the developing papillary muscles**

3D models digitally eroded along the indicated planes (yellow) to view papillary muscles (P) of the left ventricle at E14.5, E16.5 and E18.5. (Views from the right; regions enlarged are shown in red). The trabecular mesh merges to form the roots of the developing papillary muscles and this intimate arrangement is maintained throughout embryonic development.

**Figure S4. Fractal-based quantification of trabecular complexity during normal development**

The fractal dimension was calculated from HREM image data obtained with the outbred mouse strain, NIMR:Parkes. Fractal analysis is a method of quantifying complex geometric patterns in biological structures. The resulting FD is a unitless measure index of how completely the object fills space, increasing with increased structural complexity. FD was measured using a box-counting method on consecutive HREM slices of the embryo hearts in two orientations: base-to-apex and lateral-to-septal.

Panel A: Base-to-apex profile of LV and RV trabecular development (pink and grey, respectively) at stages E14.5 (LV: n = 15; RV: n = 11); E16.5 (LV: n = 13; RV: n = 11) and E18.5 (LV: n = 14; RV: n = 12). The fractal signatures of the morphological RV and LV are relatively similar at E14.5 (*P* = 0.951), but their patterns diverge as development proceeds and by E18.5 are markedly different (*P* < 0.0001).

Panel B: The LV and RV chambers from E16.5 (n = 23) and E18.5 hearts (n = 20) were digitally resliced in an orthogonal plane, from lateral-to-septal surface. At E16.5 and E18.5 note the differing lateral-to-septal patterns of FD between left and right ventricles (pink and grey, respectively) and the consistently higher FD value in the lateral wall of the RV compared to the equivalent position in the LV (*P* < 0.0001 both stages).

[Solid lines: mean FD value; shaded ribbons: 95% confidence interval. Only FD values for slices from 10-90% of base-to-apex or lateral-to-septal axis are shown].

**Figure S5. Strain differences in trabeculation detected by fractal analysis**

Comparison of base-to-apex fractal profile of LV and RV trabeculae between the outbred strain, NIMR:Parkes (grey) and the inbred strain, C57BL/6 (pink).

Panels A to C for LV data: E14.5 (n = 28); E16.5 (n = 21); E18.5 (n = 24).

Panels C to D for RV data: E14.5 (n = 19); E16.5 (n = 18); E18.5 (n = 18).

[Solid lines: mean FD value; shaded ribbons: 95% confidence interval. Only FD values for slices from 10-90% of base-to-apex are shown].

**Figure S6. Morphological abnormalities in the *Mib1* mutant heart**

3D volume renderings of E16.5 *Mib1* mutant and wildtype, sibling hearts. Models are eroded from the anterior (panels A and C) to give a four-chamber view and from the dorsal side (panels B and D) at the level of the developing aortic valve. Regions highlighted are shown (red boxes). Note the grossly aberrant trabecular arrangement, thin ventricular wall and ventricular septal defect (arrow) in the *Mib1* mutant (panel C). Wildtype left and right atria have normal pectinate muscle morphology while there is near-complete effacement in *Mib1* mutants (compare panels A and C, highlighted). The developing aortic valve in the *Mib1* mutant is abnormal, in this case the left and non-coronary leaflets (highlight panel D, asterisks) lying well above a reduced sized right leaflet. Compare with the normal trifoliate (left, right and non-coronary) leaflet arrangement (highlight panel B, labels L, R and N, respectively).

**Figure S7. Fractal analysis of *Mib1* mutant hearts**

Base-to-apex and lateral-to-septal FD plots comparing E16.5 *Mib1* mutant (*Mib1*^flox/flox^; *cTnT-cre*) hearts (pink) with wildtype littermates (grey). Note that for both left and right ventricles, fractal analysis detects significant and consistent patterns of variation between mutant and control hearts.

[Solid lines, mean; shaded ribbons, 95% confidence intervals].
