# Supplementary figures and images for: Morphogenesis of myocardial trabeculae in the mouse embryo
Source: J Anat. 2016 Mar 29;229(2):314–25. doi: 10.1111/joa.12465 (PMC4948049; doi:10.1111/joa.12465)

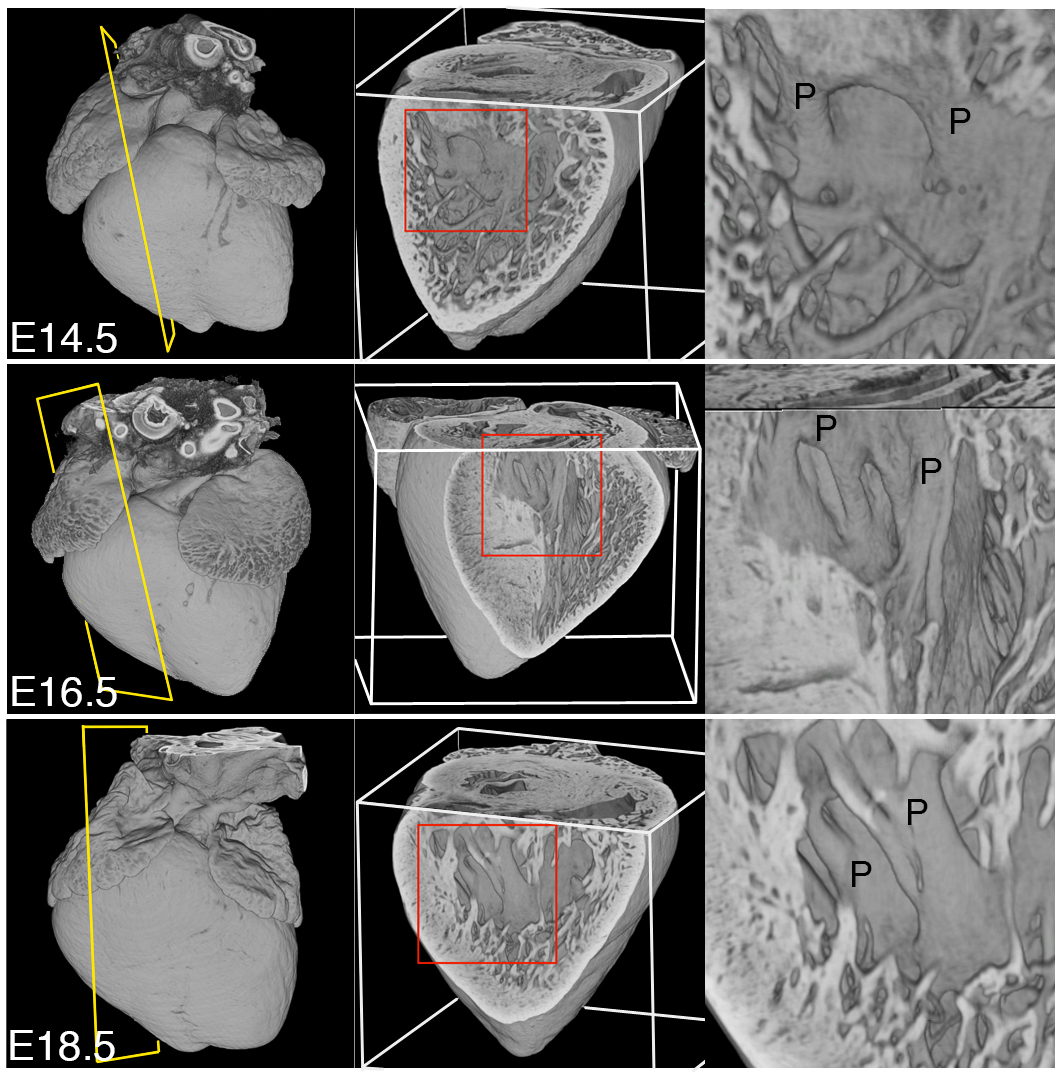

Supplement: Supplementary file 2 — Fig. S1. Papillary muscles of the right ventricle. [file JOA-229-314-s002.jpg]

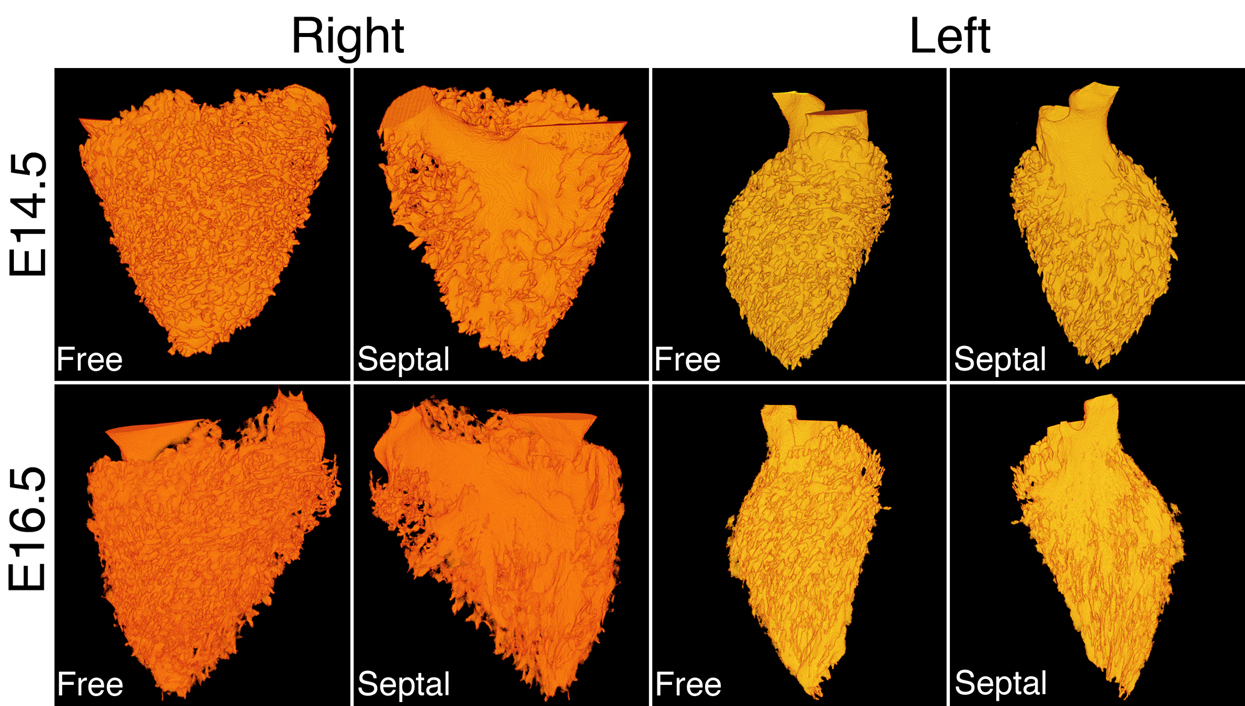

Supplement: Supplementary file 3 — Fig. S2. Volumetric models of right and left ventricular lumens. [file JOA-229-314-s003.jpg]

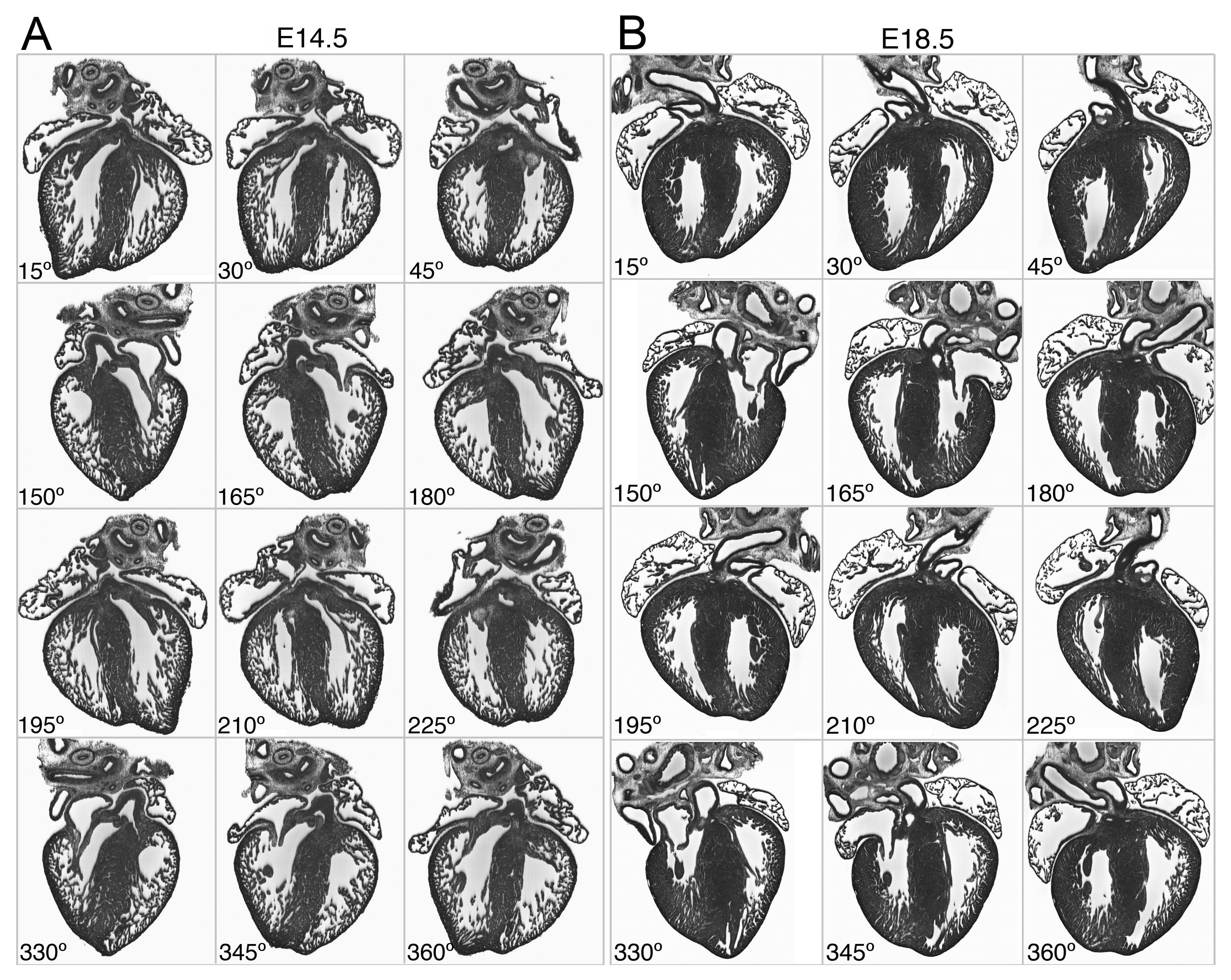

Supplement: Supplementary file 4 — Fig. S3. Comparing E16.5 wildtype hearts across two strains. [file JOA-229-314-s004.jpg]

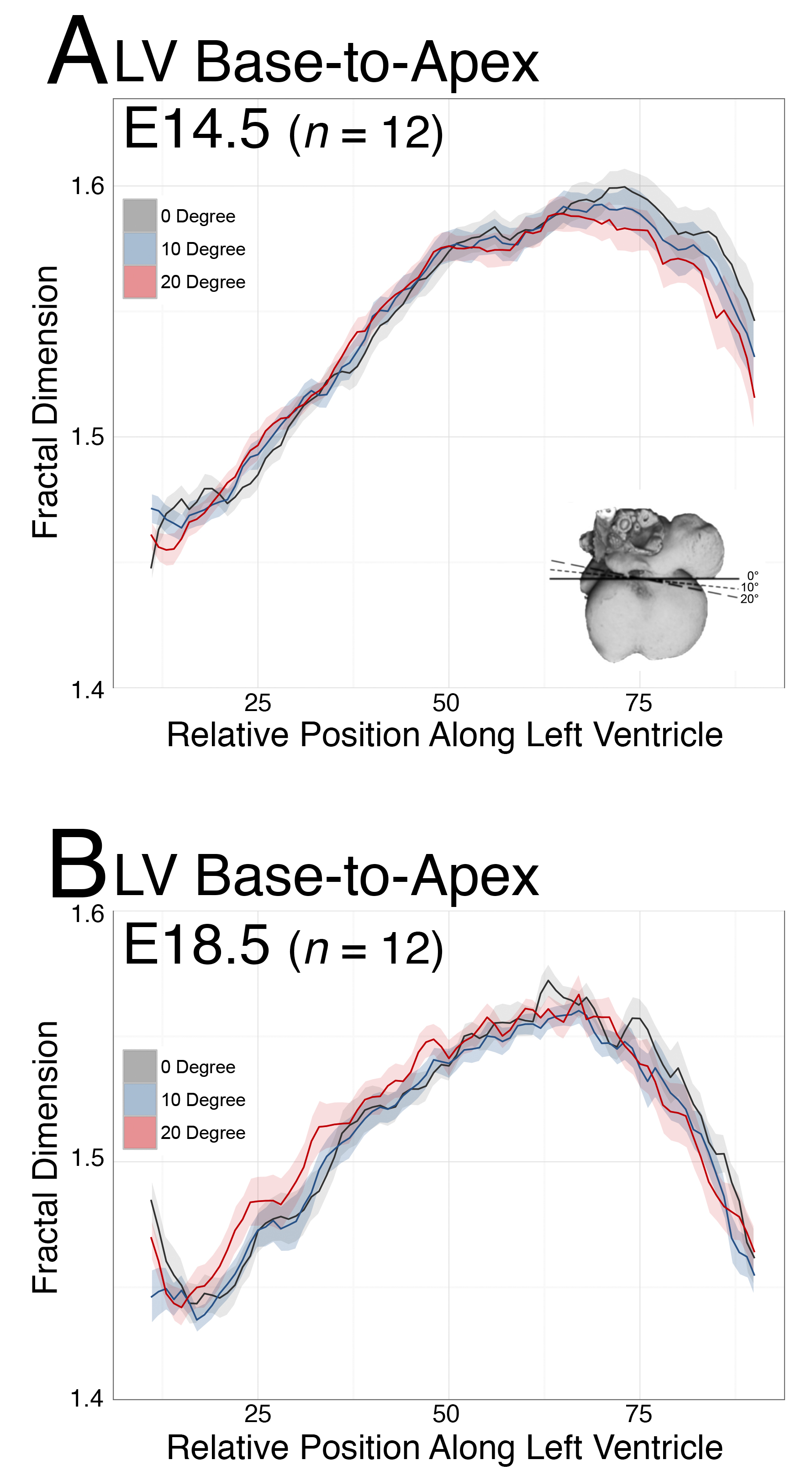

Supplement: Supplementary file 5 — Fig. S4. Effect of relative section plane on fractal dimension profile. [file JOA-229-314-s005.jpg]

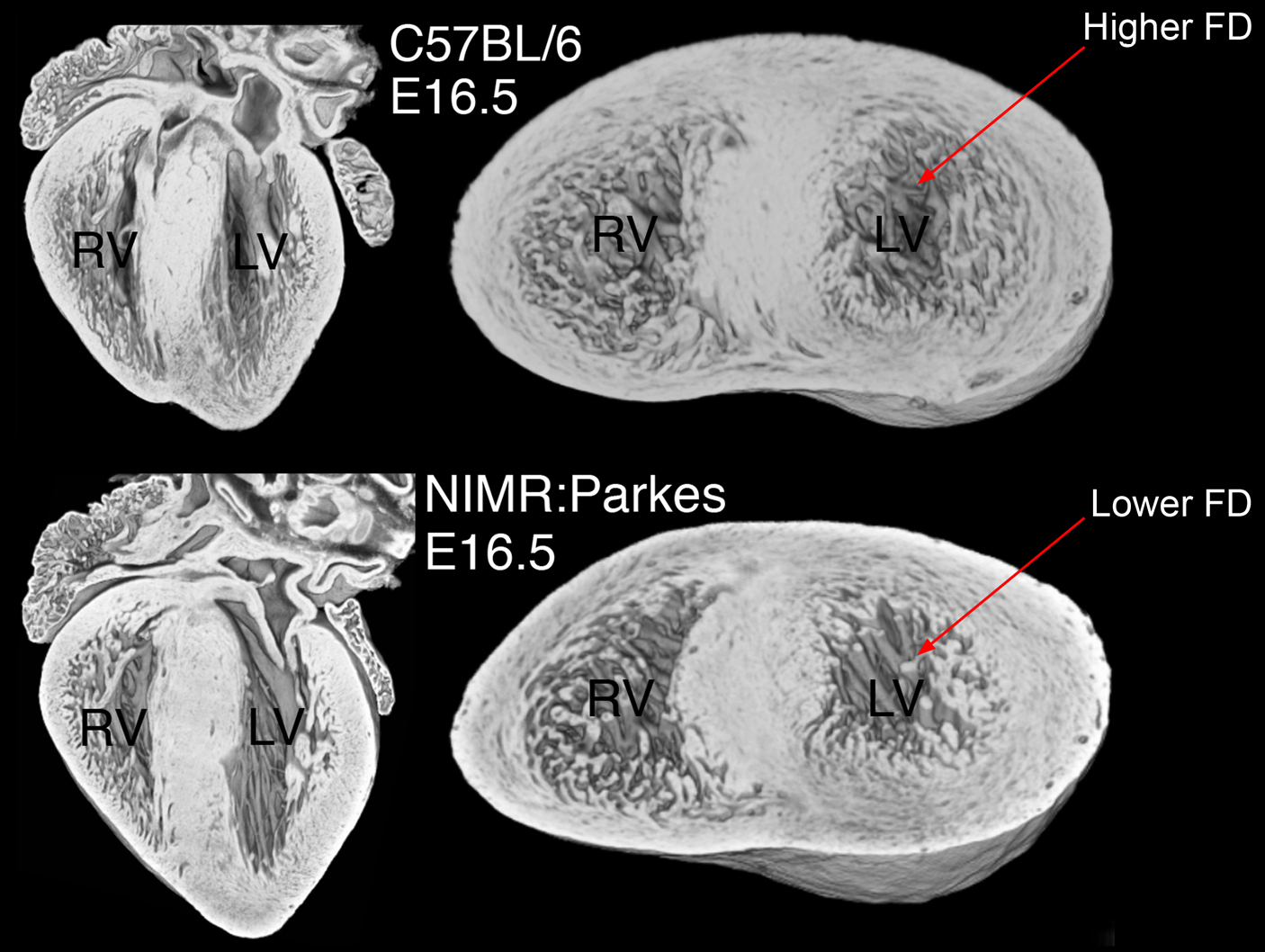

Supplement: Supplementary file 6 — Fig. S5. 3D models of an E16.5 Mib1 mutant (Mib1flox/flox; cTnT‐cre) embryo heart and a wildtype sibling (antero‐lateral views). [file JOA-229-314-s006.jpg]

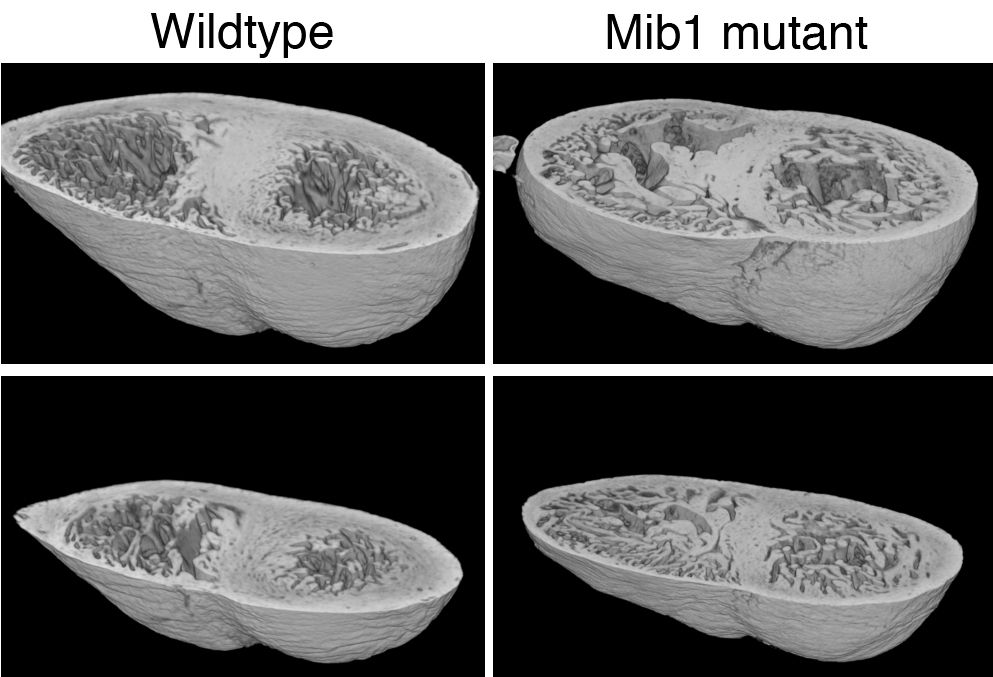

Supplement: Supplementary file 7 — Fig. S6. Comparison of HREM section images through the atria of an E16.5 Mib1 mutant (Mib1flox/flox; cTnT‐cre) embryo heart and a wildtype sibling. [file JOA-229-314-s007.jpg]

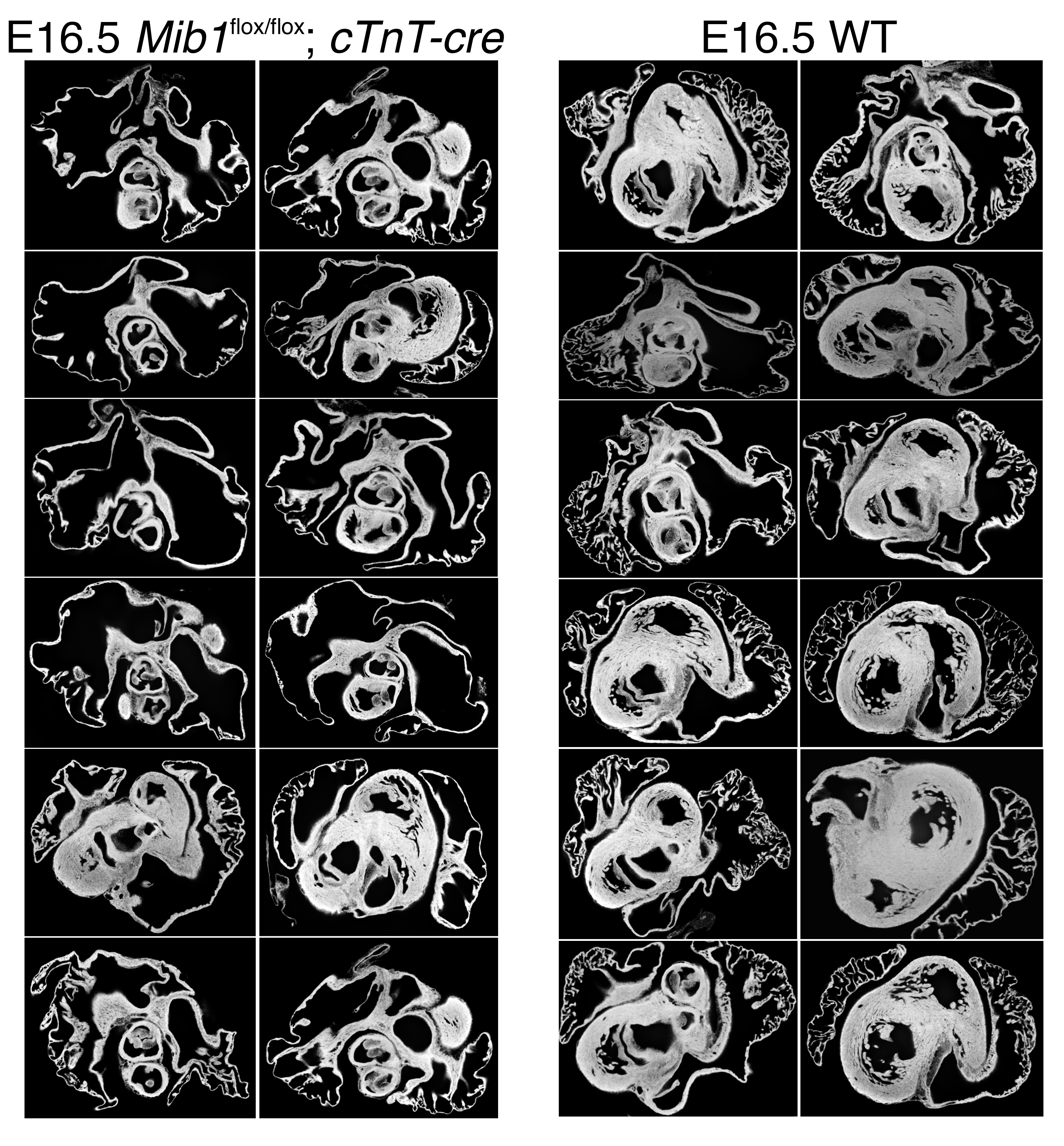

Supplement: Supplementary file 8 — Fig. S7. Virtual images generated from E14.5 and E18.5 HREM datasets (Panels A and B, respectively; NIMR:Parkes strain). [file JOA-229-314-s008.jpg]
