# Supplementary material for: Morphogenesis of myocardial trabeculae in the mouse embryo
Source: J Anat. 2016 Mar 29;229(2):314–25. doi: 10.1111/joa.12465 (PMC4948049; doi:10.1111/joa.12465)
Supplement: Supplementary file 11 [file JOA-229-314-s011.docx]

**SUPPLEMENTARY MATERIAL FOR:**

**Morphogenesis of myocardial trabeculae in the mouse embryo**

**AUTHORS**

Gabriella Captur MD PhD MRCP MSc,^1,2^ Robert Wilson PhD,^3^ Michael F Bennett BSc,^3^ Guillermo Luxán PhD,^4^ Arthur Nasis MD PhD,^5^ José Luis de la Pompa MD PhD,^4^ James C Moon MD MRCP^1,2^ & Timothy J Mohun PhD^3^

**INSTITUTIONS**

1. Institute of Cardiovascular Science, University College London, London, United Kingdom

2. The Barts Heart Centre, Barts Health NHS Trust, London, United Kingdom

3. The Francis Crick Institute Mill Hill Laboratory, The Ridgeway, Mill Hill, London, United Kingdom

4. Intercellular Signalling in Cardiovascular Development & Disease Laboratory, Centro Nacional de Investigaciones Cardiovasculares (CNIC), Melchor Fernández Almagro 3, 28029 Madrid, Spain

5. Monash Cardiovascular Research Centre, MonashHEART, Monash University, Clayton, Australia

**METHODS**

Removal of blood from embryo hearts

In order to minimise retention of blood in embryo hearts, harvested embryos were first agitated in phosphate buffered saline (PBS) solution at 37°C containing heparin for approximately 15 minutes (min), umbilical vessels being repeatedly clipped to allow blood to be pumped out. Potassium chloride was then added (final 50mM) to ensure that hearts arrested in diastole. Hearts (including attached lungs and thymus) were then isolated, washed briefly in fresh PBS and after removal of at least one lung lobe, samples were fixed for 30 min in fresh 4% paraformaldehyde at 4°C. To remove remaining blood within the heart chambers, samples were then washed in repeated changes of distilled water over 30-60min at room temperature with constant agitation (roller). The resulting osmotic shock lysed any remaining blood within the heart chambers without any alteration to heart structure as assessed by histology. After overnight fixation in 4% paraformaldehyde (4°C), hearts were dissected away from associated lung, thymus and pericardial tissue prior to dehydration and embedding in methacrylate resin (Mohun et al, 2012). Samples were positioned during embedding to ensure relatively reproducible base-to-apex sectioning during the HREM imaging process.

Fractal analysis of HREM images

HREM images were converted from 8 bit greyscale to binary and divided to separate left and right ventricular chambers using Osirix (Rosset et al, 2004) software 3D segmentation. Datasets were subsampled to yield approximately 100 images for each ventricular chamber. Luminal contours were then extracted from binary images using MATLAB® (R2012b (The MathWorks Inc., Natick, MA, USA), with in-house code for Otsu binarization (Otsu et al, 1979), a region-filling batch action that converted the image background to white, data inversion and a final edge-detection.

For fractal analysis, background pixels were set to black to permit box-counting of white foreground pixels only with smoothing filters disabled. A bounding box oriented with the *x, y* axes of the image plane was applied to each digital image to determine the relative size (pixel diameter) of the largest grid box in calculating the series of sampling sizes and scale for box counting. We determined a scaling rule for the relationship between box count and box size, following on from the assumption that these corresponded to detail and scale (ɛ) according to the equation: $\boldsymbol{FD=}\boldsymbol{lim}_{\boldsymbol{\varepsilon\to0}} \left[ {\log\boldsymbol{N\varepsilon}}/{\log\boldsymbol{\varepsilon}} \right]$, where the limit is found as the slope of the regression line for the plot of the relationship between change in count, with change in grid calibre. We replicated the analysis on the same edge image 4 times, using the same series of grid calibres but 4 different and randomly determined coordinates for starting grid positions. The final FD for any single ventricular slice was calculated as the mean of these 4 values. Results to the nearest third decimal place were automatically labelled and parsed into a comma separated variable summary data file that underwent statistical analysis. Plots of FD profile were obtained for images covering 10-90% of the image stack to minimise artefacts arising from the presence of valve leaflets or the compact ventricular wall.

Testing the effect of image plane on FD measurements

To test whether small variations in precise image plane might compromise inter-sample comparisons, we compared 2D FD plots for samples before and after digital reslicing with a 10° and 20° tilt in section plane. We used a two-factor fully cross-factored ANOVA model to test for differences. Results for the LV at both E14.5 and E18.5 (Fig. S4) show that the fractal method is robust to such variations in section plane. Comparing tilted planes with the original, at E14.5 *P* values are 0.230 and 0.066 for 10° and 20°, respectively; at E18.5, the equivalent values are 0.658 and 0.220. Similar reliability was found with the RV (data not shown).

REFERENCES FOR SUPPLEMENTARY METHODS

Mohun, T.J., Weninger, W.J., 2012. Embedding embryos for high-resolution episcopic

microscopy (HREM). Cold Spring Harb. Protoc. 6, 678–680.

Otsu, N., 1979. A threshold selection method from gray-level histograms. IEEE Trans. Syst.

Man. Cybern. 9, 62–66.

Rosset, A., Spadola, L., Ratib, O., 2004. OsiriX: an open-source software for navigating in

multidimensional DICOM images. J. Digit. Imaging. 17, 205–216.

**SUPPLEMENTARY FIGURE LEGENDS**

**Supplementary Figure 1. Papillary muscles of the right ventricle.**

3D models obtained with HREM data from embryonic hearts at E14.5, E16.5 and E18.5 (NIMR:Parkes strain). Models have been digitally eroded along the indicated planes (yellow) to view papillary muscles (P) of the right ventricle. (Views from the right; regions enlarged are shown in red). As in the left ventricle (see Fig. 3), the trabecular mesh in the RV merges to form the roots of the developing papillary muscles and this intimate arrangement is maintained throughout embryonic development.

**
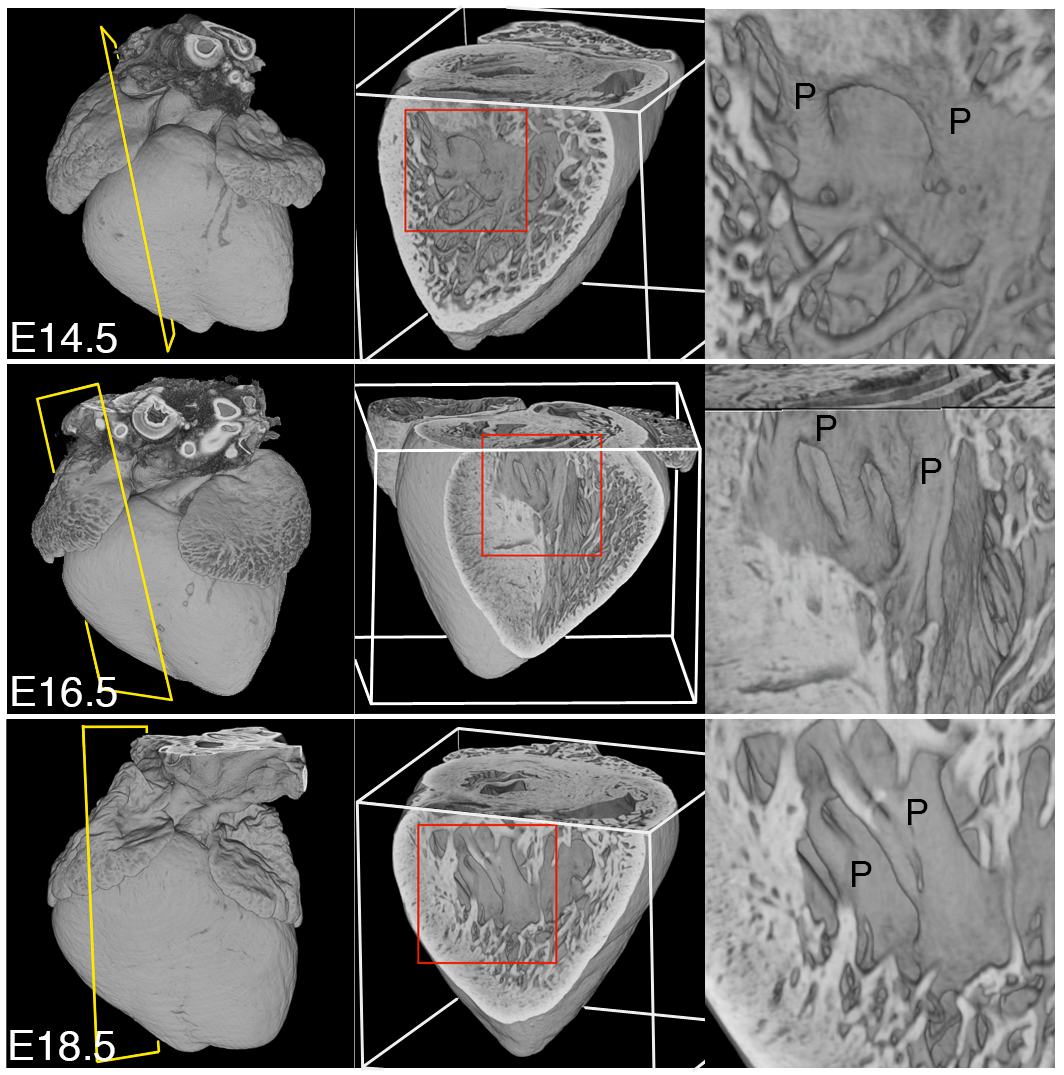
**

**Supplementary Figure 2. Volumetric models of right and left ventricular lumens.**

Volumetric models of right and left ventricular lumens derived from HREM data for embryonic hearts (NIMR:Parkes strain) at E14.5 and E16.5. Panels show the inner surfaces of the free (lateral) and septal walls. (See also Movie S1).

**
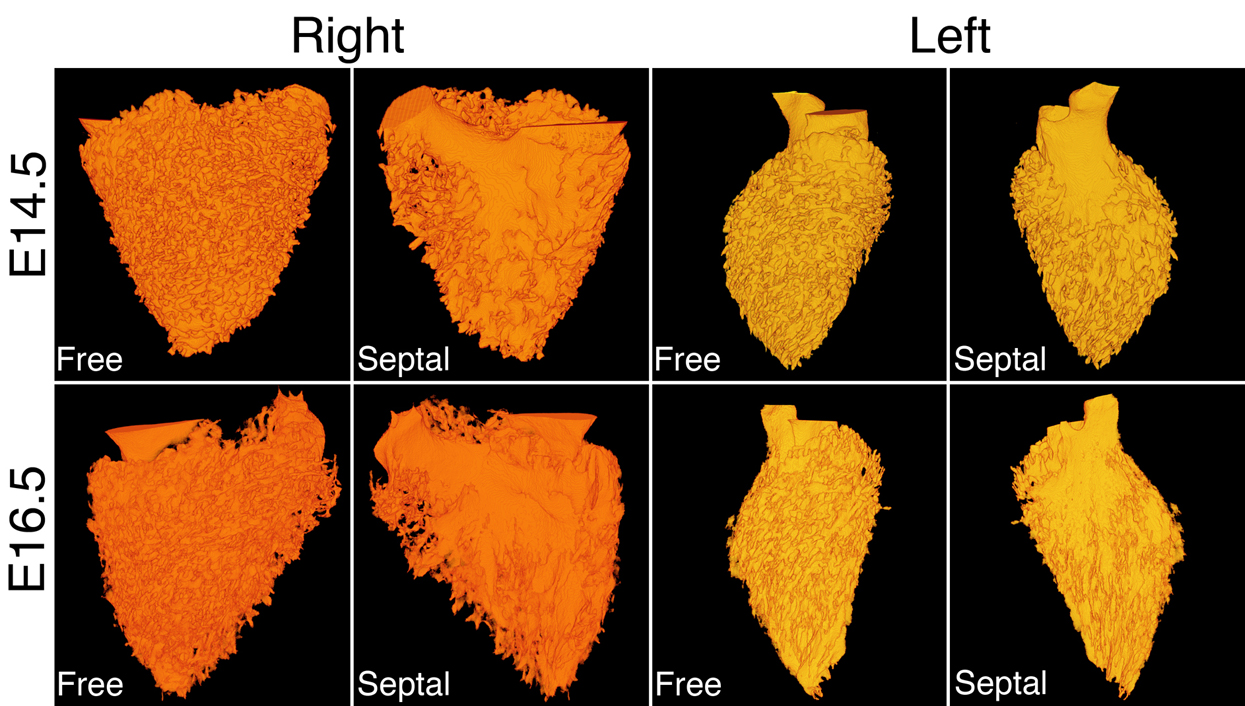
**

**Supplementary Figure 3. Comparing E16.5 wildtype hearts across two strains.**

3D models of E16.5 wildtype hearts from the outbred strain NIMR:Parkes and inbred strain, C57BL/6. Models are eroded to provide and anterior four-chamber view (left) and a short axis view of the ventricular apex (right). Note the apparent similarity in degree of trabecular complexity, an observation confounded by the more sensitive quantitative measure provided by fractal analysis (see Fig. 5).

**
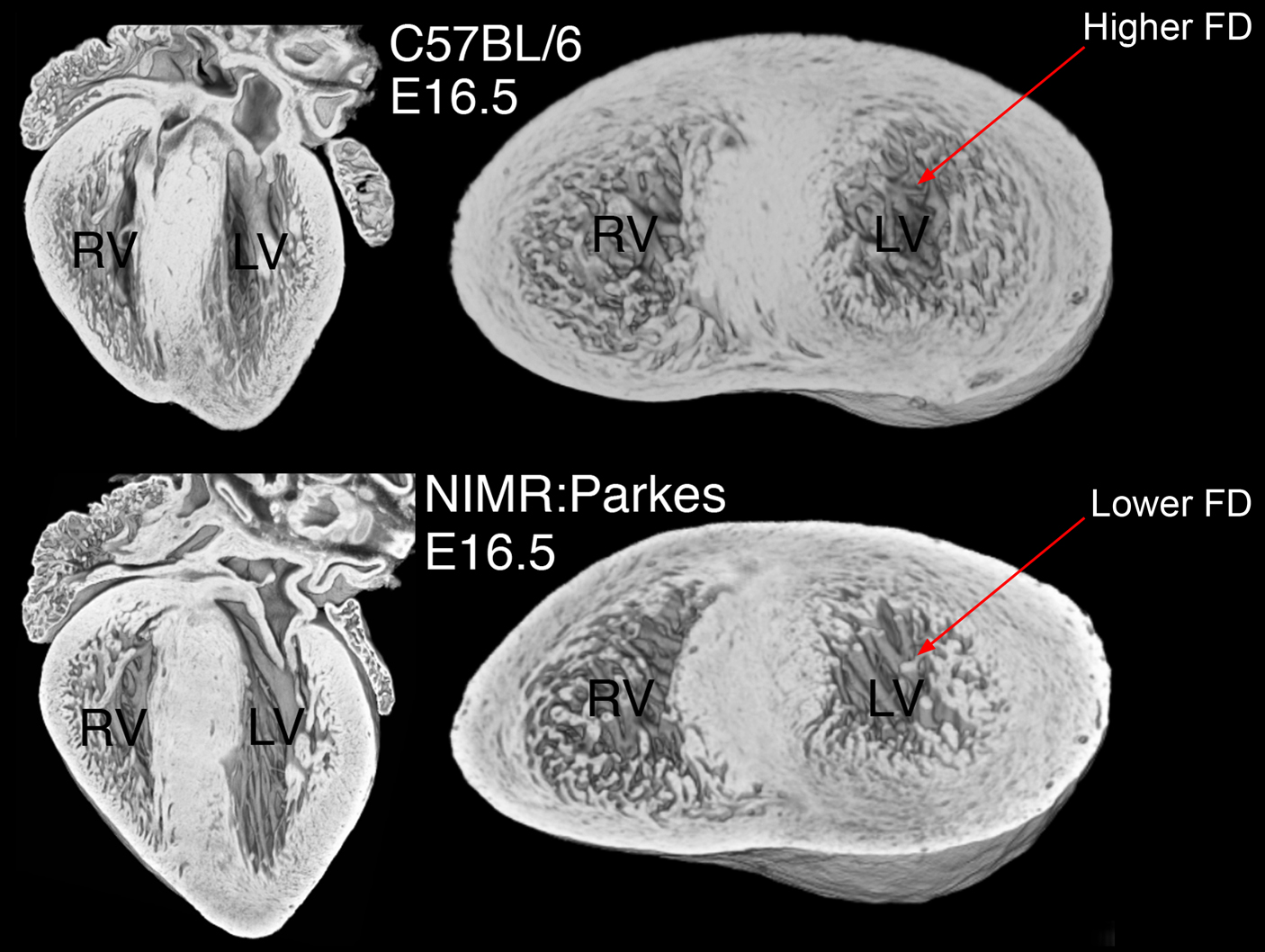
**

**Supplementary Figure 4.** **Effect of relative section plane on fractal dimension profile.**

Original section data for NIMR:Parkes strain hearts (E14.5 and E18.5) was compared with profiles after digital resectioning in 10º and 20º planes.

**
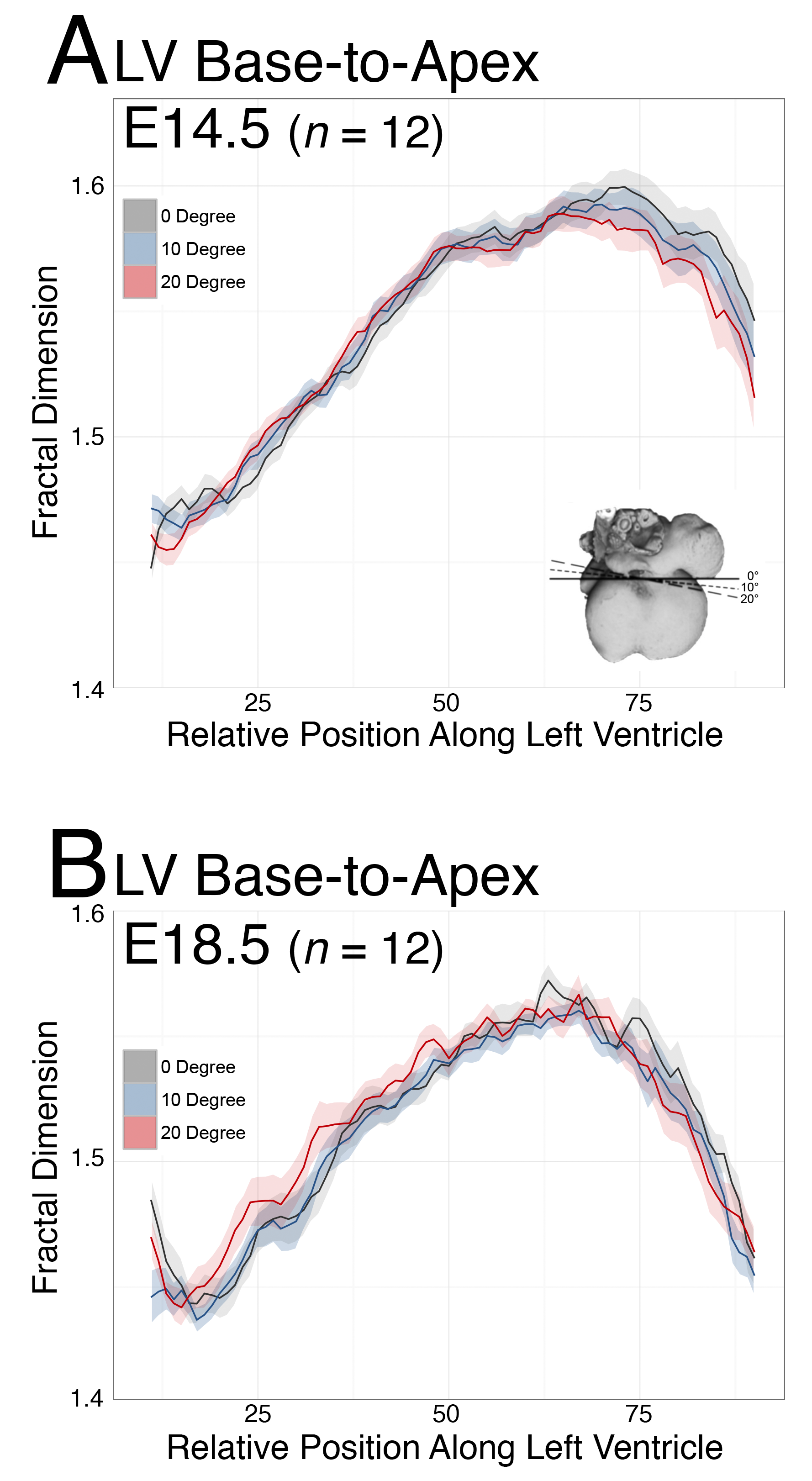
**

**Supplementary Figure 5.** **3D models of** **an E16.5 *Mib1* mutant** **(*Mib1*^flox/flox^; *cTnT-cre*) embryo heart and a wildtype sibling (antero-lateral views).**

Models are eroded to approximately two thirds and three quarters of the base-to-apex axis. Note the altered morphology of the trabecular network of the mutant, most pronounced in the right chamber and the almost complete occlusion of the apical lumen.

**
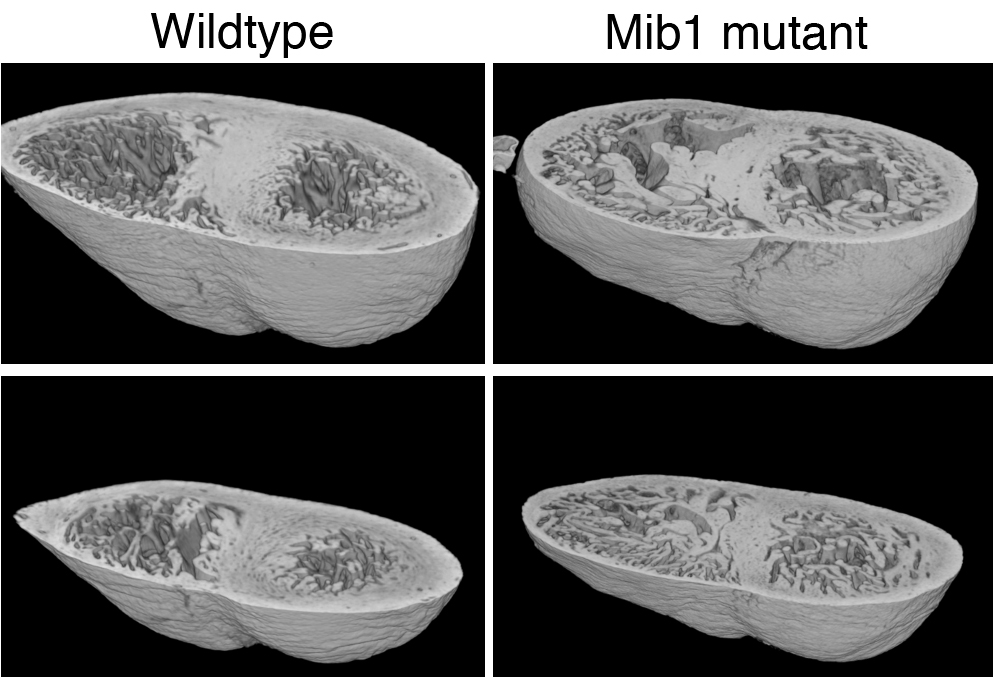
**

**Supplementary Figure 6.** **Comparison of HREM section images through the atria of** **an E16.5 *Mib1* mutant (*Mib1*^flox/flox^; *cTnT-cre*) embryo heart and a wildtype sibling.**

Note the dramatic loss of pectinate muscle from the atrial appendages of the mutant heart, resulting in a much smoother luminal surface of both left and right atria.

**
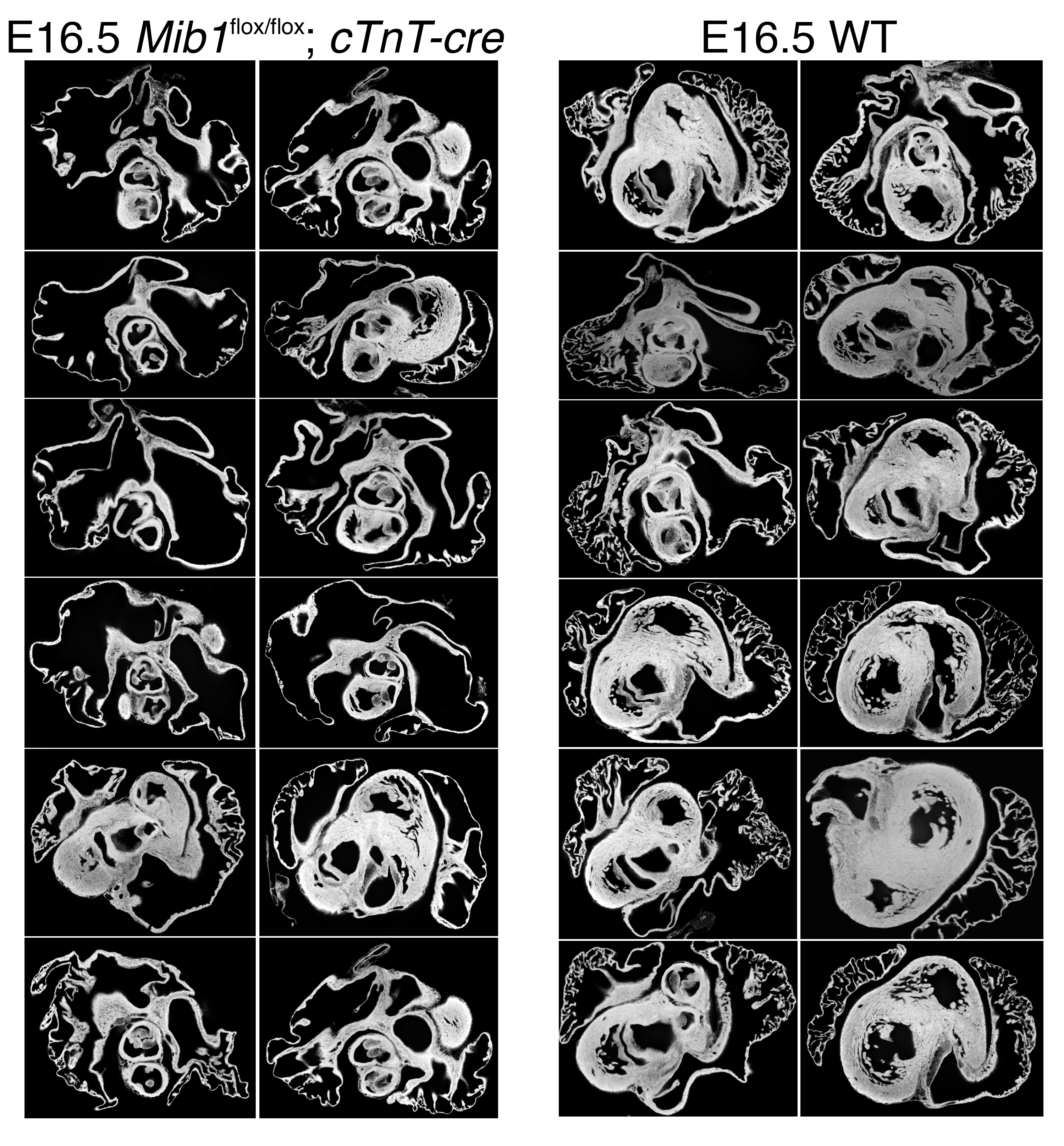
**

**Supplementary Figure 7.** **Virtual images generated from E14.5 and E18.5 HREM datasets (Panels A and B, respectively; NIMR:Parkes strain).**

Each panel comprises a radial series of four-chamber views, centred on an axis of rotation through the interventricular septum, images produced by successive 15º rotation. Note how the relative and absolute thickness of the trabecular and compact layers of each ventricle vary at different points of the ventricular wall and between different planes of section.

**
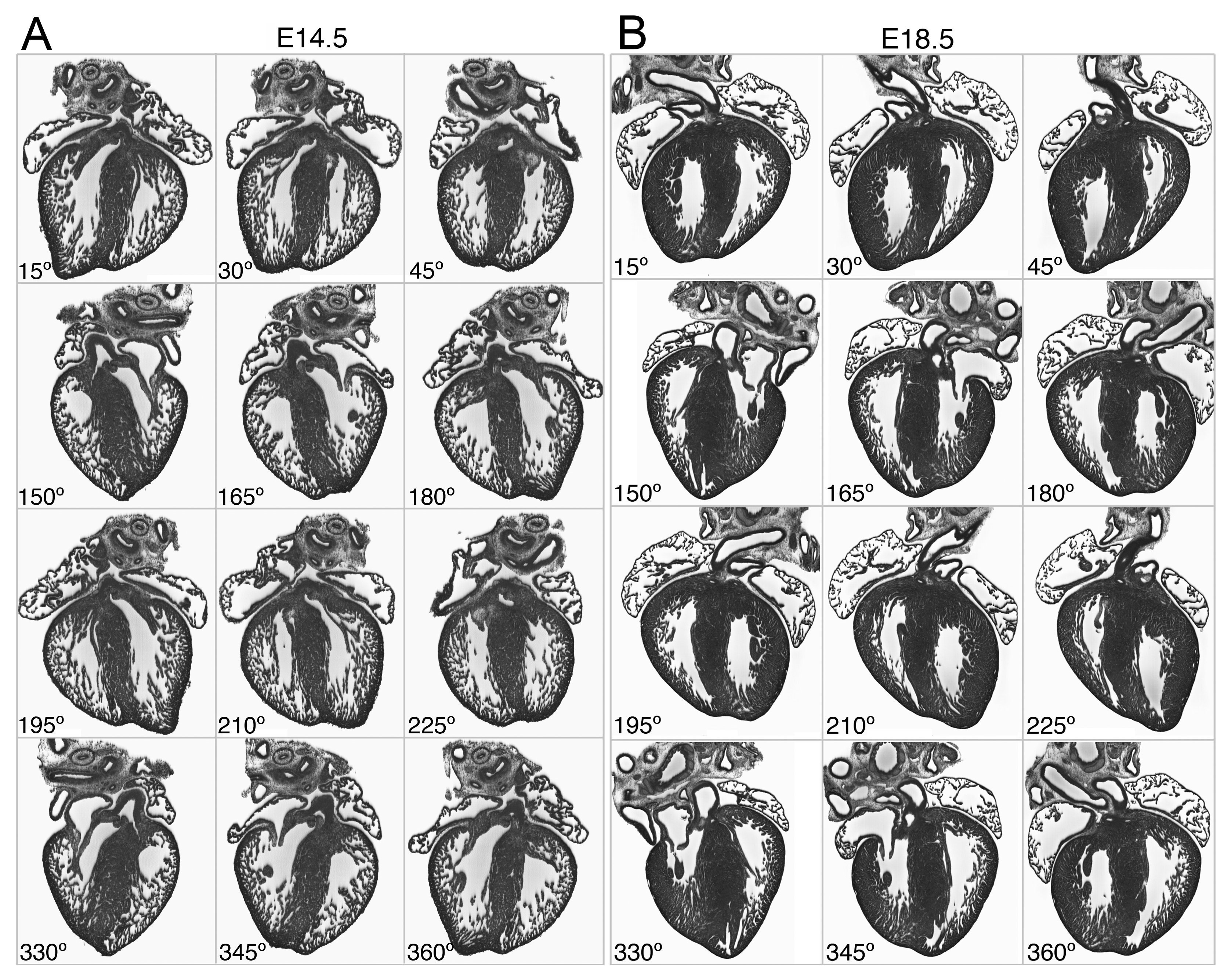
**

**SUPPLEMENTARY VIDEOS**

**Supplementary Movie 1. Ventricular luminal models of embryonic wildtype mouse heart at E14.5.**

Volumetric models of right (orange) and left (yellow) ventricular lumens derived from HREM data for embryonic hearts (NIMR:Parkes strain) at E14.5. Initially viewing each free wall side, the digital casts rotate to show their septal wall surfaces.

**Supplementary Movie 2. HREM image stack of an E16.5 *Mib1* mutant embryo heart.**

HREM image stack of an E16.5 *Mib1* mutant (*Mib1*^flox/flox^; *cTnT-cre*) embryo heart, beginning in the mid-ventricular region and showing successive images in apex-to-base direction, highlighting the bicuspid structure of the aortic valve.
